# Supplementary material for: The importance of non-pharmaceutical interventions during the COVID-19 vaccine rollout
Source: PLoS Comput Biol. 2021 Sep 10;17(9):e1009346. doi: 10.1371/journal.pcbi.1009346 (PMC8457458; doi:10.1371/journal.pcbi.1009346)
Supplement: S1 Text — In this supplementary material we present additional analyses and extensions of our model. We also run extensive sensitivity analysis on the parameters used in the main text. (PDF) [file pcbi.1009346.s001.pdf]

Supplementary Material for *The importance of non-pharmaceutical interventions during the COVID-19 vaccine rollout*

September 3, 2021

Contents

1 Epidemic Model **2**

1.1 Phase space exploration . . . 2

1.2 Constant Rate Model . . . 4

1.3 R_0 calculation . . . 5

1.4 Vaccine Efficacy . . . 8

1.5 Comparison of vaccination strategies . . . 9

1.6 Vaccine Hesitancy . . . 11

1.7 Demographic . . . 12

2 Behavioural Parameters **15**

2.1 Behavioural Parameters: α and γ . . . 15

2.2 Sensitivity analysis: r . . . 18

2.3 Sensitivity analysis: γ . . . 19

2.4 Double Behavioural Rate Model: α_S and α_V . . . 20

3 Calibrated Model **21**

3.1 Posterior Distributions . . . 21

3.2 Robustness of Vaccination Strategies to Behavioural Change . . . 22

3.3 Real Vaccination Rollout . . . 23

3.4 Reopening Scenarios . . . 24

1 Epidemic Model

1.1 Phase space exploration

In Fig 1A we explore the phase space of the behavioural parameters (α, γ) of the model. We set for the six countries $R_0 = 1.15$, 0.5% of initially infected individuals, and 10% of immune individuals, $r_V = 1\%$, $VE_S = 70\%$ and VE_{Symp} such that $VE = 90\%$. After setting the initial conditions, we let the model evolve, individually for each country, for one year exploring a grid of the parameters α and γ. This allows us to observe the phase space of parameters regulating the behavioural transitions. In particular, for each (α, γ) pair we compute the relative deaths difference due to vaccines and behaviour change. As stated in the main text, the relative deaths difference is the fraction of deaths averted with respect to a baseline without vaccine and behavioural response. For the countries of focus, we consider two different values of the parameter r ($r = 1.3$, 1.5) which defines the increase in infection risk for individuals relaxing preventive behaviours. The obtained relative deaths difference varies from a maximum of 0.86 to a minimum of 0.49. This indicates that, in our simulations in the best scenario about 86% of deaths are averted thanks to the vaccine rollout. However, this potential gain reduces to around 49% in the worst case, with a potential waste of 37% of the benefit brought by the vaccine in terms of reduced mortality. Since α and γ are the only varying parameter in these simulations, such a reduction is only attributable to the relaxation of NPIs from the individuals. More in detail, across the different settings considered, a common pattern emerges. For α fixed, as γ grows we observe progressively an increase in the relative deaths difference. Indeed, if the population reacts promptly, non-compliant individuals turn back to COVID-safe behaviours, and the fraction of averted deaths benefits from this behaviour. On contrary, for a fixed γ an increase of α induces a stronger behavioural response causing more deaths otherwise avoided thanks to the vaccination. Furthermore, we observe that, for a given pair of the behavioural parameters, the fraction of averted deaths is lower when $r = 1.5$ with respect to $r = 1.3$. Indeed, in this case non-compliant individuals expose themselves to a higher risk of infection.

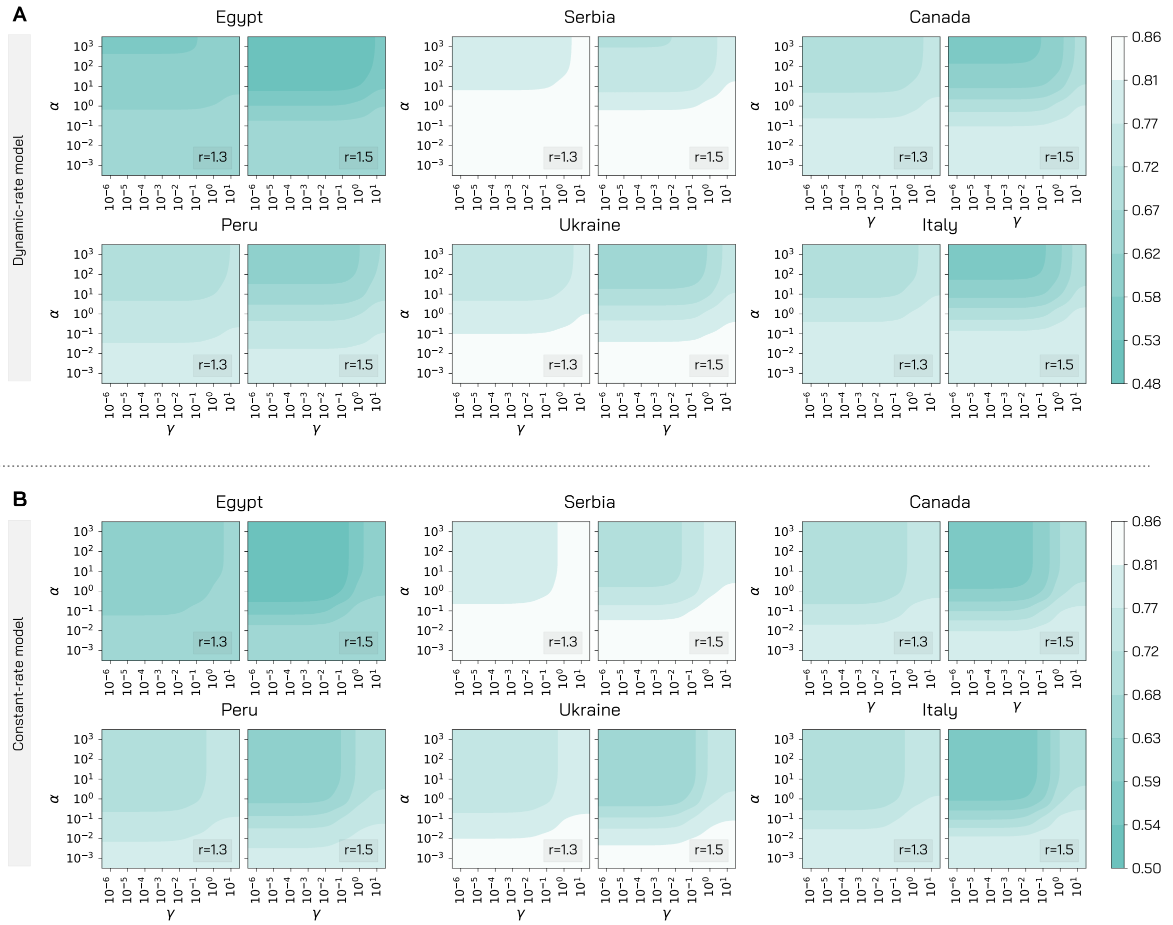

Fig 1. Impact of model parameters governing the behavioural response. For the six countries, we explore different values of α and γ in terms of fraction of averted deaths with respect to a baseline without vaccine and no behavioural response (i.e. $r_V = 0\%$, $\alpha, \gamma = 0$). We consider two different values of the parameter r (1.3 and 1.5), we set $r_V = 1\%$ and $VE_S = 70\%$ ($VE = 90\%$), and we employ vaccine strategy 1. In panel A we consider the dynamic-rate mechanism to regulate behavioural transitions (presented in the main text), while in panel B we use the constant-rate model.

1.2 Constant Rate Model

In the main text, we presented results only for the dynamic rate model in which the behavioural transitions are modulated by the fraction of vaccinated population and the number of deaths per $100,000$ in the previous time step. In Fig 1B, we report the behavioural parameters space exploration for a simpler constant rate model, in which transitions from and towards the non-compliant compartments are regulated by constant parameters. Over a grid of (γ, α) pairs, we explore the fraction of averted deaths with respect to a baseline simulation without vaccine (and thus no behaviour change triggered by the vaccination). For comparison, we display also the results for the dynamic rate model used in the main text in Fig 1A. As expected, the overall behaviour of the model is confirmed. For a fixed γ, the fraction of averted deaths reduces for increasing values of α, hinting that a stronger behavioural response causes an additional reduction of the benefit brought by the vaccine. Conversely, for a fixed α, the fraction of averted deaths increases for increasing values of γ. Indeed, in these cases non-compliant individuals turn back faster to COVID-safe behaviour.

1.3 R_0 calculation

We compute the basic reproduction number R_0 of the model proposed using the Next Generation Matrix method [1]. For simplicity, we derive the R_0 without the compartments of the vaccinated. Indeed, we imagine that the vaccination starts after that cases have increased and thus does not affect the initial stage of the epidemic. We consider the $4K$ equations that describe the evolution in time of the number of infected individuals L, P, A, and I:

$$\begin{aligned}\frac{dL_k}{dt} &= \lambda_k S_k + r\lambda_k S_k^{NC} - \epsilon L_k \\ \frac{dP_k}{dt} &= \epsilon L_k - \omega P_k \\ \frac{dA_k}{dt} &= \omega f P_k - \mu A_k \\ \frac{dI_k}{dt} &= \omega(1-f)P_k - \mu I_k\end{aligned} \tag{1}$$

Where $\lambda_k = \beta \sum_{k'=1}^{K} C_{kk'} \frac{I_{k'}+I_{k'}^V+\chi(P_{k'}+A_{k'})}{N_{k'}}$ is the force of infection for age group k. In matrix notation:

$$\begin{bmatrix}\frac{dL_1}{dt}\\ \vdots \\ \frac{dL_K}{dt}\\ \frac{dP_1}{dt}\\ \vdots\\ \frac{dP_K}{dt}\\ \frac{dA_1}{dt}\\ \vdots\\ \frac{dA_K}{dt}\\ \frac{dI_1}{dt}\\ \vdots\\ \frac{dI_K}{dt}\end{bmatrix} = \begin{bmatrix}\lambda_1 S_1 + r\lambda_1 S_1^{NC}\\ \vdots\\ \lambda_K S_K + r\lambda_K S_K^{NC}\\ 0\\ \vdots\\ 0\\ 0\\ \vdots\\ 0\\ 0\\ \vdots\\ 0\end{bmatrix} - \begin{bmatrix}\epsilon L_1\\ \vdots\\ \epsilon L_K\\ \omega P_1 - \epsilon L_1\\ \vdots\\ \omega P_K - \epsilon L_K\\ \mu A_1 - \omega f P_1\\ \vdots\\ \mu A_K - \omega f P_K\\ \mu I_1 - \omega(1-f)P_1\\ \vdots\\ \mu I_K - \omega(1-f)P_K\end{bmatrix} \tag{2}$$

$$\begin{bmatrix}\frac{d\theta_1}{dt}\\ \vdots\\ \frac{d\theta_K}{dt}\\ \frac{d\theta_{K+1}}{dt}\\ \vdots\\ \frac{d\theta_{2K}}{dt}\\ \frac{d\theta_{2K+1}}{dt}\\ \vdots\\ \frac{d\theta_{3K}}{dt}\\ \frac{d\theta_{3K+1}}{dt}\\ \vdots\\ \frac{d\theta_{4K}}{dt}\end{bmatrix} = \begin{bmatrix}F_1\\ \vdots\\ F_K\\ 0\\ \vdots\\ 0\\ 0\\ \vdots\\ 0\\ 0\\ \vdots\\ 0\end{bmatrix} - \begin{bmatrix}V_1\\ \vdots\\ V_K\\ V_{K+1}\\ \vdots\\ V_{2K}\\ V_{2K+1}\\ \vdots\\ V_{3K}\\ V_{3K+1}\\ \vdots\\ V_{4K}\end{bmatrix} \tag{3}$$

Then, we define the disease free equilibrium (DFE) for age group k as:

$$(S_k, S_k^{NC}, L_k, P_k, A_k, I_k, R_k) = (N_k, 0, 0, 0, 0, 0, 0) \tag{4}$$

Indeed, the behavioural dynamics starts only after the beginning of the vaccination campaign. We also assume that $R_k << N_k$. We define the two matrices F and V as follows: $F_{ij} = \frac{dF_i}{d\theta_j}\big|_{DFE}$ and $V_{ij} = \frac{dV_i}{d\theta_j}\big|_{DFE}$. These can be written as:

$$F=\begin{bmatrix} 0 & \cdots & 0 & \beta\frac{N_1C_{11}\chi}{N_1} & \cdots & \beta\frac{N_1C_{1K}\chi}{N_K} & \beta\frac{N_1C_{11}\chi}{N_1} & \cdots & \beta\frac{N_1C_{1K}\chi}{N_K} & \beta\frac{N_1C_{11}}{N_1} & \cdots & \beta\frac{N_1C_{1K}}{N_K} \\ \vdots & \ddots & \vdots & \vdots & \ddots & \vdots & \vdots & \ddots & \vdots & \vdots & \ddots & \vdots \\ 0 & \cdots & 0 & \beta\frac{N_KC_{K1}\chi}{N_1} & \cdots & \beta\frac{N_KC_{KK}\chi}{N_K} & \beta\frac{N_KC_{K1}\chi}{N_1} & \cdots & \beta\frac{N_KC_{KK}\chi}{N_K} & \beta\frac{N_KC_{K1}}{N_1} & \cdots & \beta\frac{N_KC_{KK}}{N_K} \\ 0 & \cdots & 0 & 0 & \cdots & 0 & 0 & \cdots & 0 & 0 & \cdots & 0 \\ \vdots & \ddots & \vdots & \vdots & \ddots & \vdots & \vdots & \ddots & \vdots & \vdots & \ddots & \vdots \\ 0 & \cdots & 0 & 0 & \cdots & 0 & 0 & \cdots & 0 & 0 & \cdots & 0 \end{bmatrix} \tag{5}$$

$$V=\begin{bmatrix} \epsilon & \cdots & 0 & 0 & \cdots & 0 & 0 & \cdots & 0 & 0 & \cdots & 0 \\ \vdots & \ddots & \vdots & \vdots & \ddots & \vdots & \vdots & \ddots & \vdots & \vdots & \ddots & \vdots \\ 0 & \cdots & \epsilon & 0 & \cdots & 0 & 0 & \cdots & 0 & 0 & \cdots & 0 \\ -\epsilon & \cdots & 0 & \omega & \cdots & 0 & 0 & \cdots & 0 & 0 & \cdots & 0 \\ \vdots & \ddots & \vdots & \vdots & \ddots & \vdots & \vdots & \ddots & \vdots & \vdots & \ddots & \vdots \\ 0 & \cdots & -\epsilon & 0 & \cdots & \omega & 0 & \cdots & 0 & 0 & \cdots & 0 \\ 0 & \cdots & 0 & -\omega f & \cdots & 0 & \mu & \cdots & 0 & 0 & \cdots & 0 \\ \vdots & \ddots & \vdots & \vdots & \ddots & \vdots & \vdots & \ddots & \vdots & \vdots & \ddots & \vdots \\ 0 & \cdots & 0 & 0 & \cdots & -\omega f & 0 & \cdots & \mu & 0 & \cdots & 0 \\ 0 & \cdots & 0 & -\omega(1-f) & \cdots & 0 & 0 & \cdots & 0 & \mu & \cdots & 0 \\ \vdots & \ddots & \vdots & \vdots & \ddots & \vdots & \vdots & \ddots & \vdots & \vdots & \ddots & \vdots \\ 0 & \cdots & 0 & 0 & \cdots & -\omega(1-f) & 0 & \cdots & 0 & 0 & \cdots & \mu \end{bmatrix} \tag{6}$$

The basic reproduction number is defined as $R_0=\rho(FV^{-1})$, where $\rho(\cdot)$ indicates the spectral radius. First, we compute V^{-1}. We recognize that V and F can be written in blocks as:

$$F=\begin{bmatrix} 0 & \chi\beta\tilde{C} & \chi\beta\tilde{C} & \beta\tilde{C} \\ 0 & 0 & 0 & 0 \\ 0 & 0 & 0 & 0 \\ 0 & 0 & 0 & 0 \end{bmatrix}, V=\begin{bmatrix} H & 0 & 0 & 0 \\ I & L & 0 & 0 \\ 0 & M & N & 0 \\ 0 & P & 0 & R \end{bmatrix} \tag{7}$$

Where all the block components of V are diagonal matrices, and $\tilde{C}$ is the contacts matrix weighted by the relative population in different age groups (i.e. $\tilde{C_{ij}}=\frac{N_i}{N_j}C_{ij}$). The inverse of a block matrix $\begin{bmatrix} A & 0 \\ C & D \end{bmatrix}$ can be written as $\begin{bmatrix} A^{-1} & 0 \\ -D^{-1}CA^{-1} & D^{-1} \end{bmatrix}$, where in our case $A=\begin{bmatrix} H & 0 \\ I & L \end{bmatrix}, C=\begin{bmatrix} 0 & M \\ 0 & P \end{bmatrix}, D=\begin{bmatrix} N & 0 \\ 0 & R \end{bmatrix}$. Therefore, we compute:

$$A^{-1}=\begin{bmatrix} H & 0 \\ I & L \end{bmatrix}^{-1}=\begin{bmatrix} H^{-1} & 0 \\ -L^{-1}IH^{-1} & L^{-1} \end{bmatrix} \tag{8}$$

$$D^{-1}=\begin{bmatrix} N & 0 \\ 0 & R \end{bmatrix}^{-1}=\begin{bmatrix} N^{-1} & 0 \\ 0 & R^{-1} \end{bmatrix} \tag{9}$$

$$\begin{aligned} -D^{-1}CA^{-1} &= -\begin{bmatrix} N^{-1} & 0 \\ 0 & R^{-1} \end{bmatrix}\begin{bmatrix} 0 & M \\ 0 & P \end{bmatrix}\begin{bmatrix} H^{-1} & 0 \\ -L^{-1}IH^{-1} & L^{-1} \end{bmatrix} \\ &= -\begin{bmatrix} 0 & N^{-1}M \\ 0 & R^{-1}P \end{bmatrix}\begin{bmatrix} H^{-1} & 0 \\ -L^{-1}IH^{-1} & L^{-1} \end{bmatrix} \\ &= \begin{bmatrix} N^{-1}ML^{-1}IH^{-1} & -N^{-1}ML^{-1} \\ R^{-1}PL^{-1}IH^{-1} & -R^{-1}PL^{-1} \end{bmatrix} \end{aligned} \tag{10}$$

Substituting these expressions, we can then write V^{-1}:

$$V^{-1}=\begin{bmatrix} H^{-1} & 0 & 0 & 0 \\ L^{-1}IH^{-1} & L^{-1} & 0 & 0 \\ N^{-1}ML^{-1}IH^{-1} & -N^{-1}ML^{-1} & N^{-1} & 0 \\ R^{-1}PL^{-1}IH^{-1} & -R^{-1}PL^{-1} & 0 & R^{-1} \end{bmatrix} \tag{11}$$

The next step consists in computing the product FV^{-1}:

$$FV^{-1} = \begin{bmatrix} 0 & \chi\beta\tilde{C} & \chi\beta\tilde{C} & \beta\tilde{C} \\ 0 & 0 & 0 & 0 \\ 0 & 0 & 0 & 0 \\ 0 & 0 & 0 & 0 \end{bmatrix} \begin{bmatrix} H^{-1} & 0 & 0 & 0 \\ L^{-1}IH^{-1} & L^{-1} & 0 & 0 \\ N^{-1}ML^{-1}IH^{-1} & -N^{-1}ML^{-1} & N^{-1} & 0 \\ R^{-1}PL^{-1}IH^{-1} & -R^{-1}PL^{-1} & 0 & R^{-1} \end{bmatrix}$$

$$= \beta\tilde{C} \begin{bmatrix} (-\chi\mathbb{1} + \chi N^{-1}M + R^{-1}P)L^{-1}IH^{-1} & \chi L^{-1} - \chi N^{-1}ML^{-1} - R^{-1}PL^{-1} & \chi N^{-1} & R^{-1} \\ 0 & 0 & 0 & 0 \\ 0 & 0 & 0 & 0 \\ 0 & 0 & 0 & 0 \end{bmatrix} \tag{12}$$

Finally, we are left with finding the spectral radius of FV^{-1} (i.e., its largest eigenvalue). The eigenvalue problem can be written as $det(FV^{-1} - \lambda\mathbb{1}) = 0$. Given the structure of FV^{-1}, and since we are interested in non-trivial solutions ($\lambda \neq 0$), the problem reduces to:

$$det[\beta\tilde{C}(-\chi\mathbb{1} + \chi N^{-1}M + R^{-1}P)L^{-1}IH^{-1} - \lambda\mathbb{1}] = 0 \tag{13}$$

Since N, M, R, P, L, I, and H are all diagonal we easily compute the inverses and products and simplify the expression to:

$$det\left[\beta\left(\frac{\chi}{\omega} + \frac{f\chi}{\mu} + \frac{1-f}{\mu}\right)\tilde{C} - \lambda\mathbb{1}\right] = 0 \tag{14}$$

Therefore, finding the spectral radius of FV^{-1} is equivalent to solving the eigenvalue problem for $\beta\left(\frac{\chi}{\omega} + \frac{f\chi}{\mu} + \frac{1-f}{\mu}\right)\tilde{C}$ and taking the largest eigenvalue. Finally, we have that $R_0 = \beta\left(\frac{\chi}{\omega} + \frac{f\chi}{\mu} + \frac{1-f}{\mu}\right)\rho(\tilde{C})$

1.4 Vaccine Efficacy

In the main text we let vary the rollout speed r_V and kept fixed the vaccine efficacy. Here, we repeat part of the analyses varying also their effectiveness. More in detail, we consider three vaccine efficacy $VE = 50\%, 70\%, 90\%$. We set the related $VE_S = 30\%, 50\%, 70\%$, and we adjust accordingly the VE_{Symp} using the formula $VE = 1 - (1 - VE_S)(1 - VE_{Symp})$. For these values, we represent the relative deaths difference as function of α for the six countries and the three prioritization strategies. Results are reported in Fig 2. As expected, a higher VE leads to a higher fraction of averted deaths. In the case of Italy, when $\alpha = 0$ and vaccination strategy 1 is employed, 55% of deaths are avoided when $VE = 90\%$, 44% when $VE = 70\%$, and 33% when $VE = 50\%$. When we switch on behavioural response (i.e., $\alpha > 0$), we observe similar patterns of the main text. Indeed, lower vaccine efficacy are more impacted by the relaxation of NPIs. As an example, in the case of Ukraine and vaccination strategy 1, when $VE = 90\%$ the fraction of averted deaths goes from 0.57 with $\alpha = 0$ to -0.20 with $\alpha = 10$, with a potential drop of 0.77. When instead $VE = 50\%$ these figures drop to 0.37 ($\alpha = 0$) and -1.08 ($\alpha = 10$), with an increased drop of 1.45.

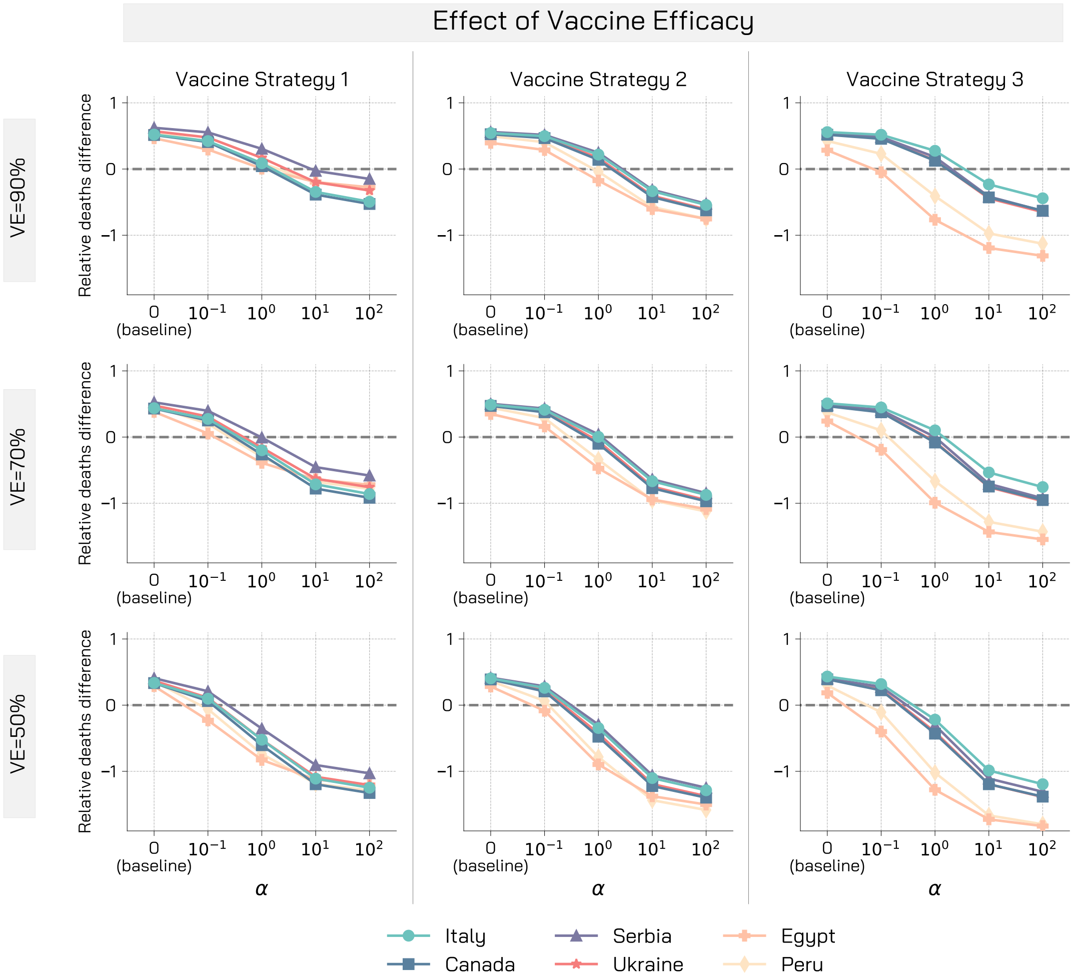

Fig 2. Relative deaths difference for different vaccine efficacy and prioritization strategies. Relative deaths difference is computed as the fraction of deaths that are avoided with a vaccine with respect to a baseline simulation without vaccine. We display results of the simulations for three vaccine efficacy and prioritization strategies. Other parameters used are $\gamma = 0.5$, $R_0 = 1.15$, $r = 1.3$, $r_V = 0.25\%$, $VE_S = 70\%$ and VE_{Symp} such that $VE = 90\%$, 0.5% of initially infected, 10% of initially immune individuals, and simulations length is set to 1 year.

1.5 Comparison of vaccination strategies

In the main text, we considered the number of deaths as a primary endpoint to evaluate the efficacy of behaviour and vaccine. In Fig 3 we compare the different vaccination strategies both in terms of averted deaths and averted infections. We set $\alpha = 0$, therefore we do not consider behaviour change. We observe that the strategy prioritizing the elderly (i.e., strategy 1), is actually the most efficient in reducing the number of deaths across the different population pyramids and contact patterns considered. The strategy prioritizing age groups $20 - 49$ (i.e., strategy 3) is the best one in reducing infections for Serbia, Ukraine, Canada, and Italy. In the case of Egypt and Peru, while strategy 3 is preferable to strategy 1 when considering the fraction of averted infections, the most efficient one in this case is the strategy that targets homogeneously the population (i.e., strategy 2). This may be due to the high contacts activity of individuals aged under 20, who are partly vaccinated since the beginning of the campaign when strategy 2 is employed.

In Fig 4, we repeat the analysis presented in the main text considering the relative infections difference instead of the relative deaths difference as an endpoint to evaluate the effects of vaccines and behaviour on the spreading. By relative infections difference, we simply intend the fraction of averted infections in the presence of a vaccine with respect to baseline without vaccine. We consider the three vaccine prioritization strategies, the three rollout speed, $r_V = 0.1\%, 0.5\%, 1\%$, and different intensity of the behavioural responses by exploring a range of α values. We observe that the most efficient strategies at reducing the number of infections are strategy 3 (for Serbia, Ukraine, Canada, and Italy) and 2 (for Egypt and Peru). Across the different countries, we observe that strategy 1 is generally the worst one in terms of averted infections and it is also more affected by stronger behavioural responses.

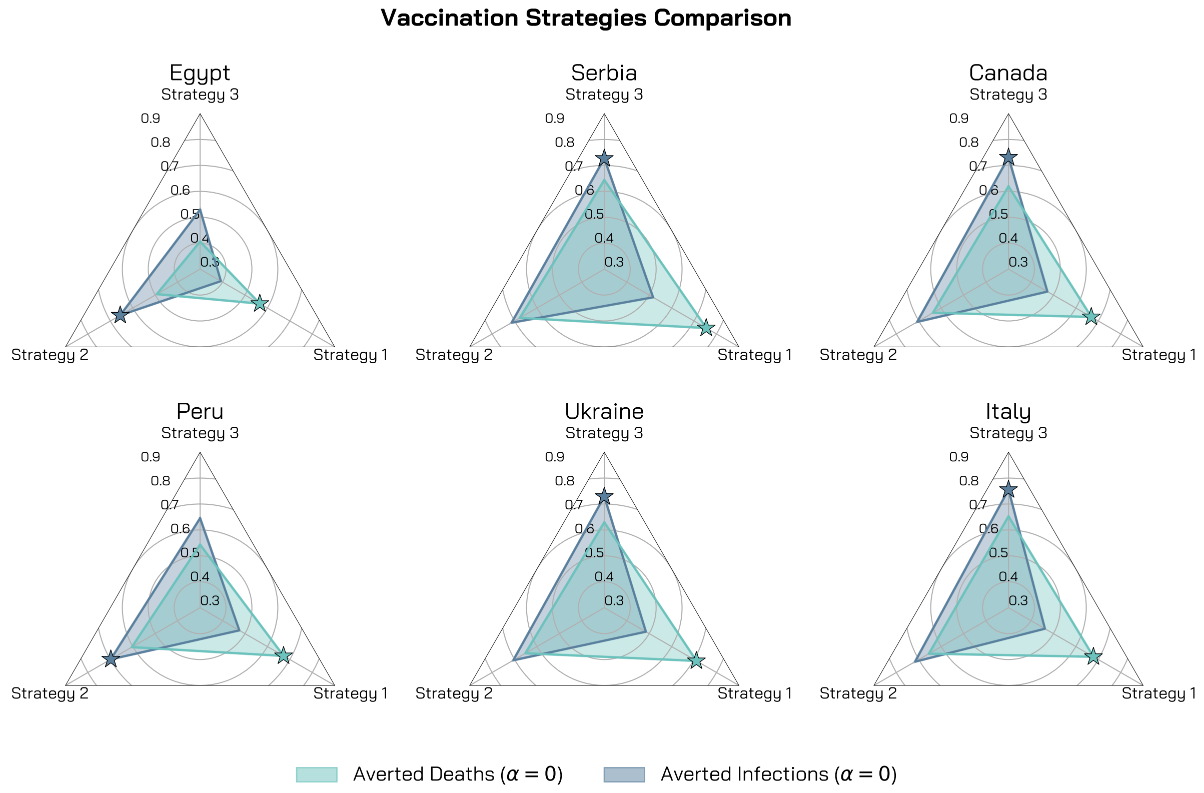

Fig 3. Comparison of different vaccination strategies. We compare the three vaccination strategies considered in terms of averted deaths and averted infections when $\alpha = 0$ with respect to a baseline without vaccine. Stars indicate best strategy. We set $\gamma = 0.5$, $R_0 = 1.15$, $r = 1.3$, $r_V = 0.5\%$, $VE_S = 70\%$ ($VE = 90\%$), 0.5% of initially infected, 10% of initially immune individuals, and simulations length is set to 1 year.

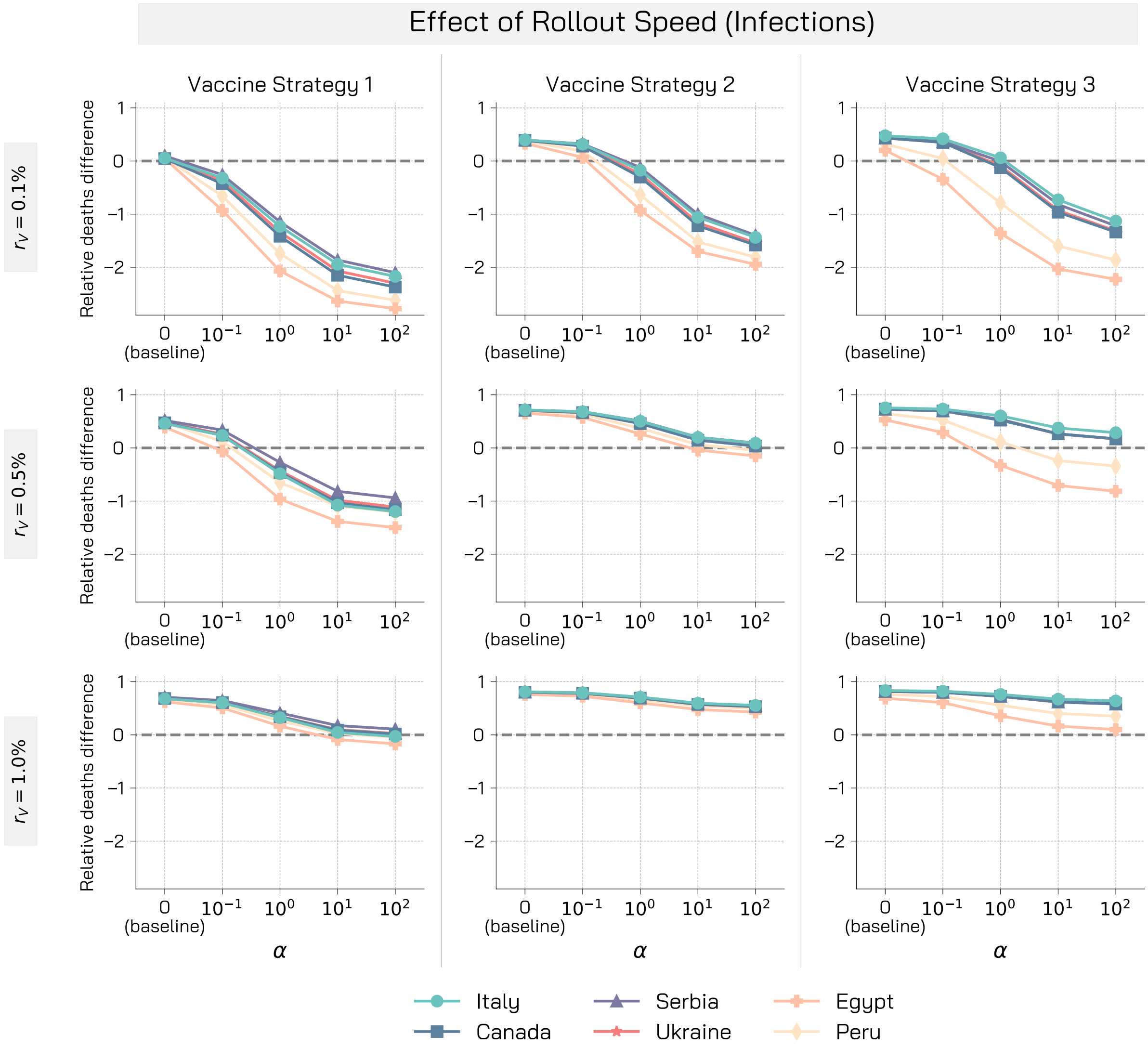

Fig 4. Relative infections difference for different rollout speed and prioritization strategies. Relative infections difference is computed as the fraction of infections that are avoided with a vaccine with respect to a baseline simulation without vaccine (and thus no behavioural response). We display results of the simulations for three vaccine efficacy and prioritization strategies. Other parameters used are $\gamma = 0.5$, $R_0 = 1.15$, $r = 1.3$, $VE_S = 70\%$ ($VE = 90\%$), 0.5% of initially infected, 10% of initially immune individuals, and simulations length is set to 1 year.

1.6 Vaccine Hesitancy

In the main text we assumed that all individuals are willing to receive the vaccine. This is an optimistic assumption, since some may decide not to get vaccinated. As noted previously, vaccine adoption is complex, and may depend on a range of individual factors [2]. As a sensitivity check here, we extend our modeling framework to account also for vaccine hesitancy. In Fig 5 we show the relative deaths difference for the six countries as function of α for three different values of percentage of the population refusing the vaccine: 0% (i.e., 100% uptake), 20% (i.e., 80% uptake), and 40% (i.e., 60% uptake). As expected, an higher fraction of the population refusing to get a vaccine results in worse outcomes measured in terms of averted deaths. In the case of Peru, when everyone gets vaccinated and $\alpha = 0$, 55% of deaths are averted with respect to a baseline without vaccines. This figure lowers to 52% and 48% when, respectively, 20% and 40% of the population decide not to get vaccinated. More interestingly, when behaviour relaxation comes into play, much wider differences are observed. Following the previous example, when $\alpha = 10$, with no vaccine hesitancy the relative deaths difference is equal to 0.09, to -0.06 with 20% and to -0.20 with 40% hesitancy.

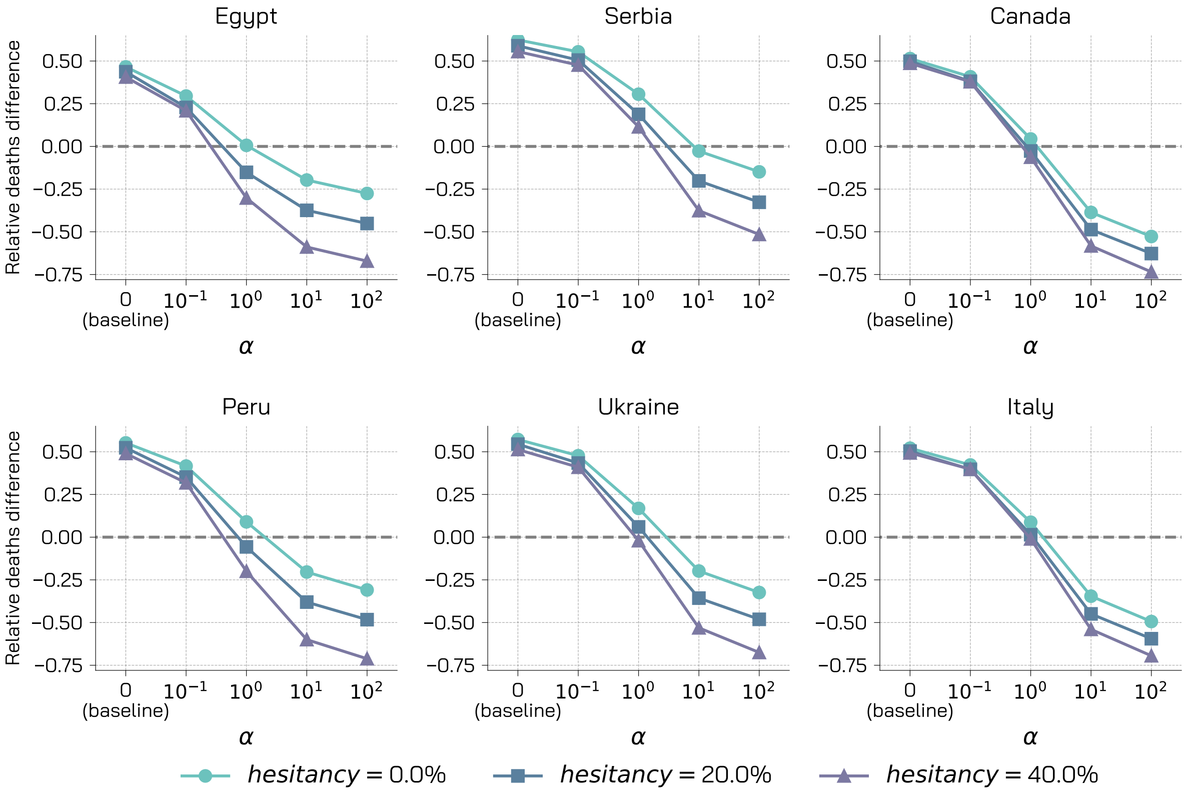

Fig 5. Relative deaths difference for different vaccine hesitancy. We represent for the different countries the relative deaths difference as function of α for three values of vaccine hesitancy (i.e., percentage of the population not willing the receive a vaccine). We consider vaccination strategy 1 aimed at reducing severity. We set $R_0 = 1.15$, $r_V = 0.25\%$, $VE_S = 70\%$ ($VE = 90\%$), $\gamma = 0.5$, 0.5% of initially infected, 10% of initially immune individuals, and simulations length is set to 1 year.

1.7 Demographic

In this section we provide additional information about the demographic of the different countries studied. In Fig 6 we show, for each country, the four layers of the contacts matrix (*school*, *work*, *home*, *other locations*). We observe how the developing economies (i.e., Egypt, Peru) and the economies in transitions (i.e., Ukraine) show a higher number of daily contacts. In Fig 7 we plot instead the fraction of people in different age groups for the six countries. As mentioned in the main text, Egypt and Peru feature a much younger population, while Italy is the country with the highest fraction of people in the 75+ age group.

Finally, we further investigate the interplay among contacts patterns, demographic pyramids and behaviour change in Fig 8. For simplicity, we consider only Egypt and Italy. They are two very dissimilar countries in terms of demographics. Indeed, as noted previously, Egypt has a younger population, higher number of contacts, and higher inter-generational mixing. For both countries we run a simulation with vaccine rollout followed by a possible relaxation of NPIs ($\alpha = 10$) and one with vaccines allocation only ($\alpha = 0$). We then compare the number of daily deaths per $100'000$ in the two simulations. In Fig 8 we see clearly that, in both simulations more deaths occur in Italy. Notice how we kept for visualization purposes two different scale of the y-axis. Indeed, at the peak nearly 5 deaths per $100'000$ are counted daily for Italy, while, in the worst case, less than 0.5 are observed in Egypt. This result is expected: the older population and the strong dependence of COVID-19 infection fatality rate from age makes Italy more fragile to the spreading. Nonetheless, we observe an interesting finding. As noted in the main text, we see that, in relative terms, behavioural response has a bigger impact in the case of Egypt. Indeed, switching on the behavioural response results in around 29% more deaths for Italy. In the case of Egypt this figure is more than doubled: about 59% deaths increase is observed when behavioural change comes into play. This comparison underlines once more the impact of socio-demographics on the behavioural dynamics and shows in more detail how a younger, more active, and more mixed population is more exposed to the risk of COVID-safe behaviour relaxation.

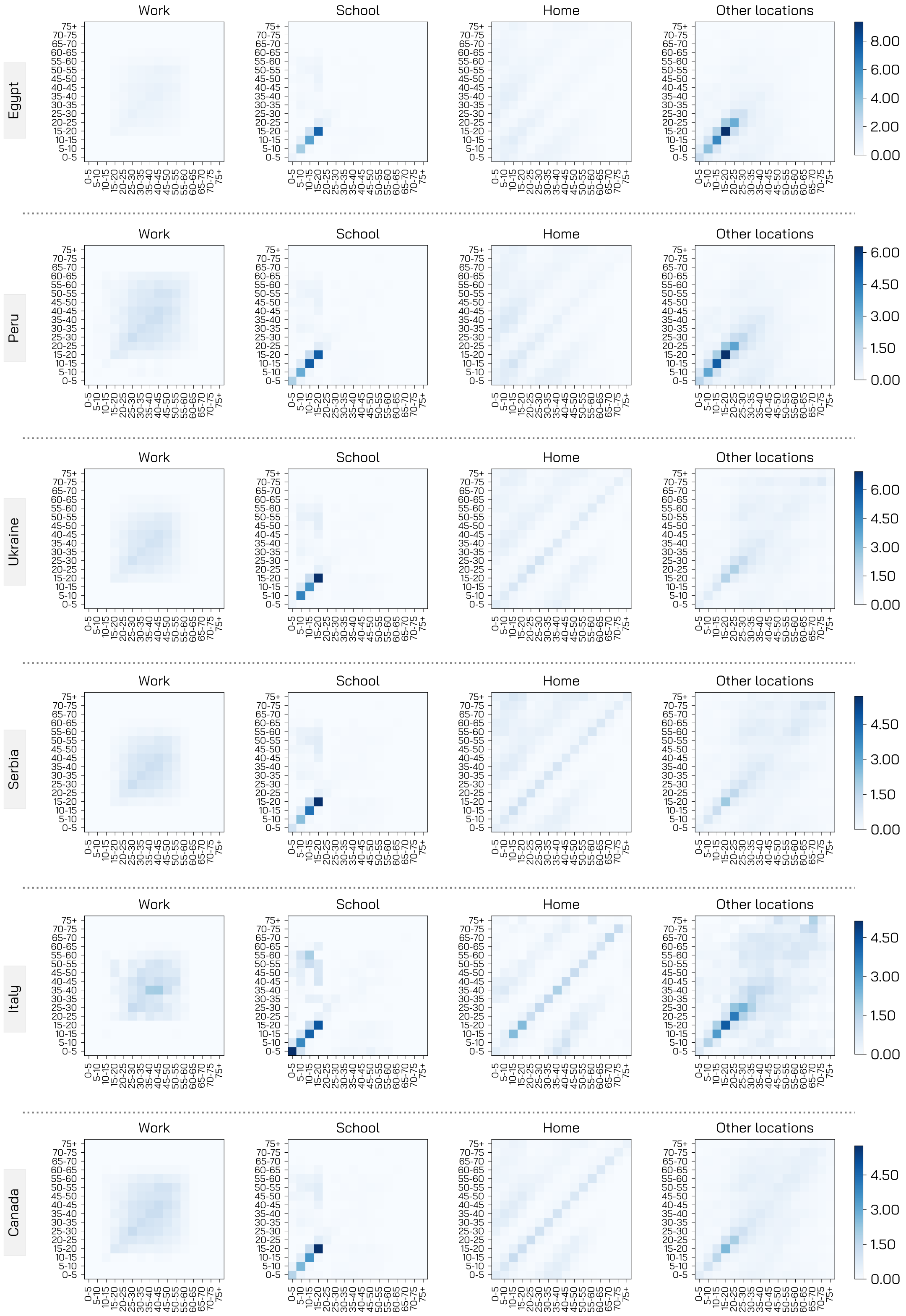

Fig 6. Contacts Matrices.

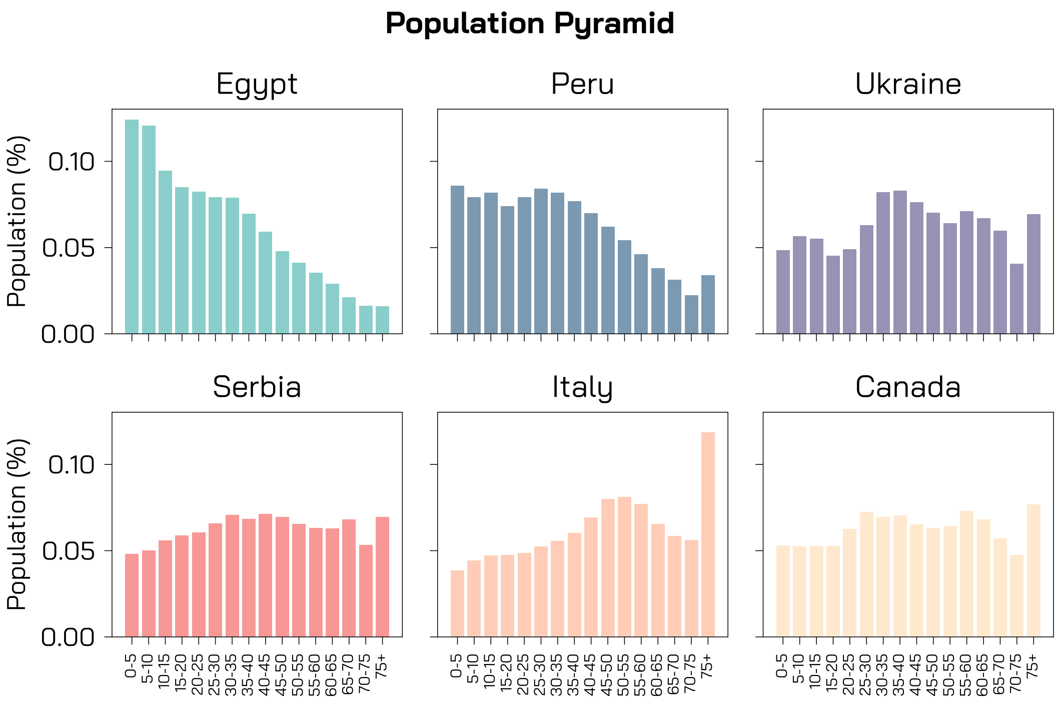

Fig 7. Population Pyramids.

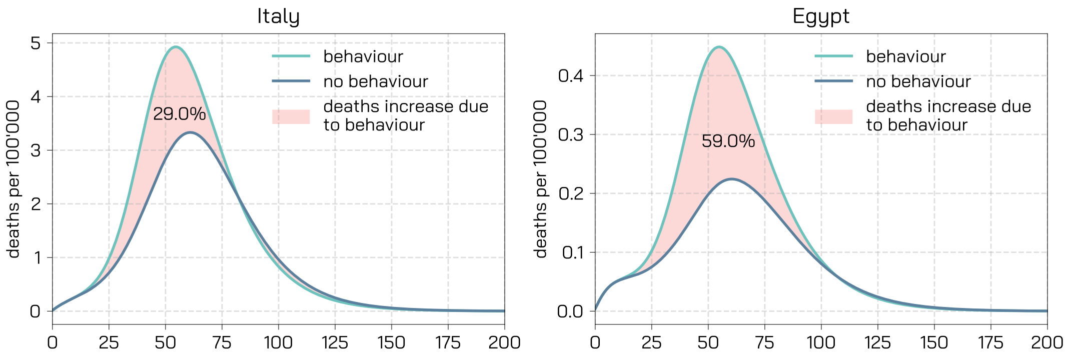

Fig 8. Effects of behaviour change: comparison between Italy and Egypt. We display daily deaths for $100'000$ for Italy and Egypt in the case of a simulation with possible behaviour relaxation ($\alpha = 10$), and one without ($\alpha = 0$). In both cases we set: $R_0 = 2.0$, $r_V = 0.25\%$, $VE_S = 70\%$ ($VE = 90\%$), $\gamma = 0.5$, $r = 1.3$, and we employ the vaccination strategy aimed at reducing severity (strategy 1).

2 Behavioural Parameters

In this section, we provide additional insights on the role and the interpretation of the behavioural parameters, as well as sensitivity analyses on the values used in the main text.

2.1 Behavioural Parameters: α and γ

To analyse in depth the role of α and γ in Fig 9 we plot the rates of the behavioural transitions for several combination of the parameters. In panel A we show the rate of the transition from compliance to non-compliance $g(\alpha) = 1 - e^{-\alpha v_t}$ as function of the fraction of the population vaccinated v_t and for a spectrum of values of α. As expected, the rates are bounded between 0 and 1 and, across the different α, $g(\alpha)$ show an increasing trend. Indeed, the higher the fraction of population vaccinated, the more probable is the transition to non-compliance. For increasing values of α, the function $g(\alpha)$ grows faster and approaches the maximum value 1 for lower values of v_t. As an example, when $v_t = 0.2$, the transition rate $g(\alpha)$ is equal to 0.02, when $\alpha = 0.1$, to 0.33 when $\alpha = 2$, and to 0.86 when $\alpha = 10$. For extreme values such as $\alpha = 100$, the growth of $g(\alpha)$ is virtually instantaneous, and the transition rate approaches 1 for very low values of v_t. This extreme case represent a collective, strong, response to the vaccine rollout in which individuals massively give up COVID-safe behaviours as soon as the vaccination start. To further understand the effect of different α, we plot as a dashed horizontal line the reference value of $g(\alpha) = 0.5$: we observe that in general for $\alpha < 1$ the transition probability is smaller than 50% when the whole population is vaccinated (i.e., $v_t = 1$). In panel B, we repeat the same analysis for the rate of the transition from non-compliance to compliance $h(\gamma) = 1 - e^{-\gamma d_t^o}$. We study it as a function of the number of observed daily deaths per $100'000$ d_t^o and for several values of γ. We use values of d_t^o between 0 and 5. As a reference, in the worst day of the Pandemic so far Italy reported about 1.6 deaths per $100'000$. The observations made previously hold also in this case: the transition rate shows an increasing trend as function of d_t^o and for increasing values of γ the growth of the function $h(\gamma)$ becomes steeper.

In Fig 10 we show, for different values of α and R_0, the temporal evolution of the fraction of non-compliant individuals ($S^{NC} + V^{NC}$), together with the fraction of vaccinated population and the number of daily deaths per $100'000$. We observe that, for higher values of α, the fraction of non-compliant grows faster. In parallel, since a stronger relaxation of NPIs leads to a larger number of infections, we also observe more deaths and less vaccinated. More in detail, especially when $\alpha = 1, 10$, we observe that after reaching a peak, the fraction of non-compliant decreases. The reason is that, following the relaxation of NPIs from a group of individuals, the number of deaths increases and as result individuals go back to safer behaviours. This can be easily seen by comparing the peak of number of deaths per $100'000$ (last row) and the local minimum in the fraction of non-compliant (first row) around $t = 80$. For higher t, the fraction of non-compliant reaches a plateau: indeed, when the number of infected goes to zero, all S and V individuals left transition to the non-compliant compartments. Interestingly, for $\alpha = 0.1$, the final fraction of non-compliant is higher. The reason is that, thanks to a milder relaxation of COVID-safe behaviours, less people get infected and therefore more move to non-compliance when the number of infected goes to zero. Finally, when R_0 is higher we observe less individuals becoming non-compliant. Indeed, more deaths occur because of the higher transmissibility, and as result individuals are less likely to abandon COVID-safe behaviours.

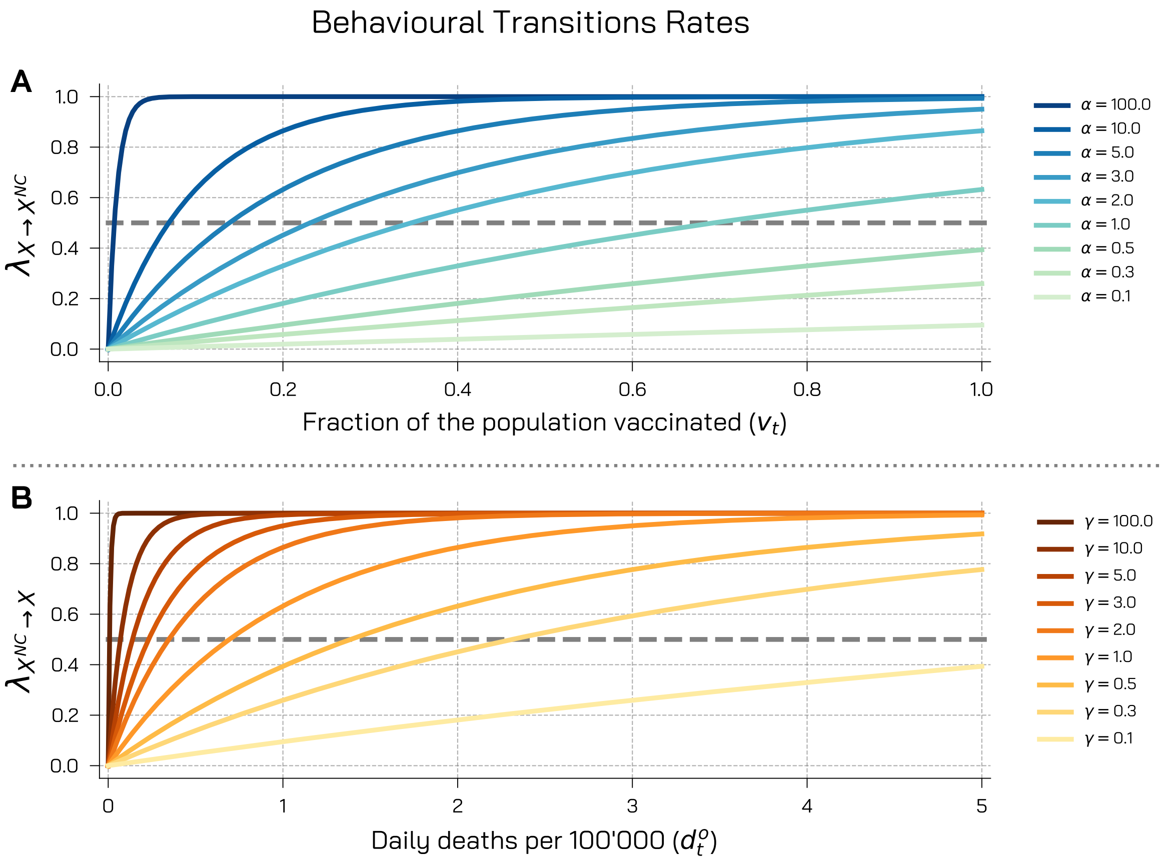

Fig 9. Behavioural transitions rates. We represent the rates of the behavioural transitions for different parameters. A) Rate of the transition from compliance to non-compliance ($g(\alpha)$) as a function of the fraction of the population vaccinated (v_t) for different values of α. B) Rate of the transition from non-compliance to compliance ($h(\gamma)$) as a function of observed daily deaths per $100'000$ (d_t^o) for different values of γ. The horizontal dashed line is placed at 0.5.

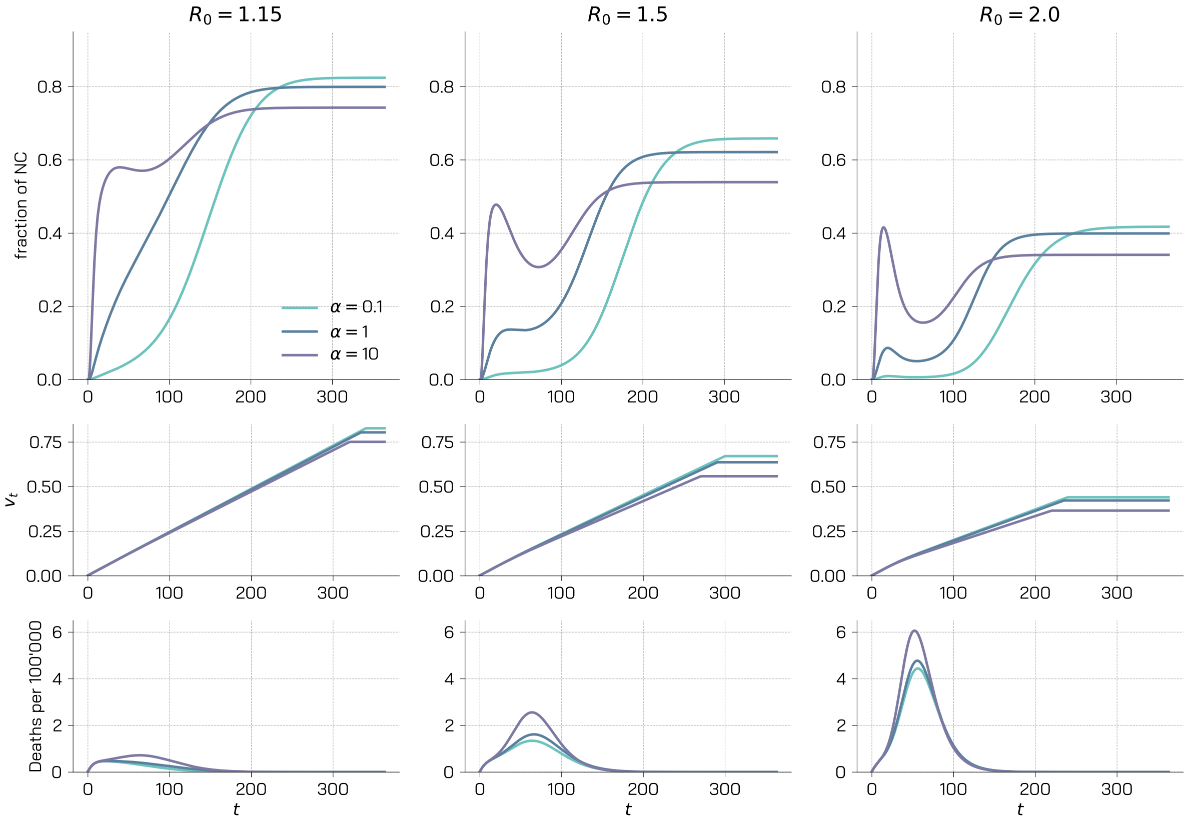

Fig 10. Fraction of non-compliant individuals in time. We represent the fraction of non-compliant individuals over time for different values of α and epidemiological conditions ($R_0 = 1.15, 1.5, 2.0$). We also represent the factors regulating the behavioural transitions, namely the fraction of vaccinated individuals in time and the number of daily observed deaths per $100'000$. We consider the population pyramid and contacts patterns of Italy, a vaccination strategy targeting the population homogeneously, and we set $VE_S = 70\%$ ($VE = 90\%$), $r_V = 0.25\%$, $\gamma = 0.5$, $r = 1.3$, 10% of initially recovered and 0.5% initially infected individuals.

2.2 Sensitivity analysis: r

In the main text we run the simulations considering $r = 1.3$. This parameter describes the increased risk of infection that non-compliant individuals (S^{NC} and V^{NC}) face when giving up COVID-safe behaviours. In the main text we justified the choice $r = 1.3$ considering the estimated effects of NPIs such as mask wearing and social distancing on COVID-19 spreading [3, 4, 5, 6]. Here, we run a sensitivity analysis to this choice in Fig 11. We display, for the six different countries of focus, the relative deaths difference as function of α for three values of r $(1.1, 1.3, 1.5)$. We consider vaccination strategy 1 (aimed at reducing severity), $r_V = 1\%$, and $VE_S = 70\%$ $(VE = 90\%)$. Overall, we observe that the patterns observed in the main text hold when changing r. Indeed, when $\alpha > 0$, the fraction of averted deaths, thanks to the vaccines diminishes. As expected, lower values of r imply smaller impact on the relative deaths difference. In the case of Italy, for example, when $\alpha = 10$, 75% of deaths are averted in the case of $r = 1.1$, 64% when $r = 1.3$, and finally 48% when $r = 1.5$.

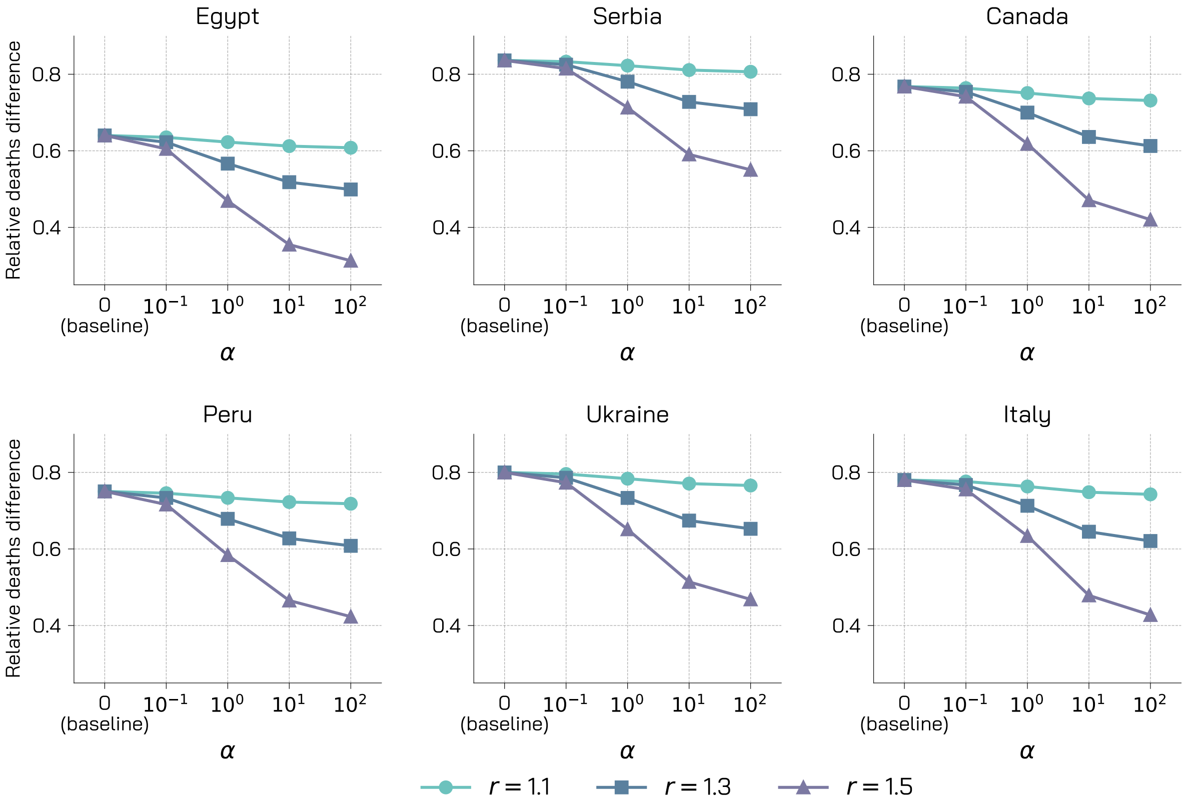

Fig 11. Relative deaths difference for different r. We represent for the different countries the relative deaths difference as function of α for three values of the behavioural parameter r $(r = 1.1, 1.2, 1.3)$. We consider vaccination strategy 1 aimed at reducing severity. We set $R_0 = 1.15$, $r_V = 1\%$, $VE_S = 70\%$ $(VE = 90\%)$, $\gamma = 0.5$, 0.5% of initially infected, 10% of initially immune individuals, and simulations length is set to 1 year.

2.3 Sensitivity analysis: γ

In the main text we generally kept constant the behavioural parameter γ ($\gamma = 0.5$) and we let vary the other parameter α. We informed the choice of γ looking at the maximum number of COVID-19 deaths observed on a single day in the different countries. In the case of Italy, for example, $\gamma = 0.5$ would have implied a 60% probability for non-compliant to go back to safer behaviours with 1000 deaths in the previous step. Here, we report results obtained for different values of γ. We consider two additional values, $\gamma = 0.05$ and $\gamma = 2$, which in the previous example would imply, respectively, a return probability of 10% and 95%. In Fig 12 we compare, for each country, the relative deaths difference for the three values of γ (0.05, 0.5, 2). We observe that, while the decreasing trend is common across the different γ considered, curves for higher values of γ are shifted upwards. In other words, when γ grows the fraction of averted deaths increases. This is expected: indeed, a higher γ implies greater awareness to deaths increase, and thus individuals go back to safer behaviours faster.

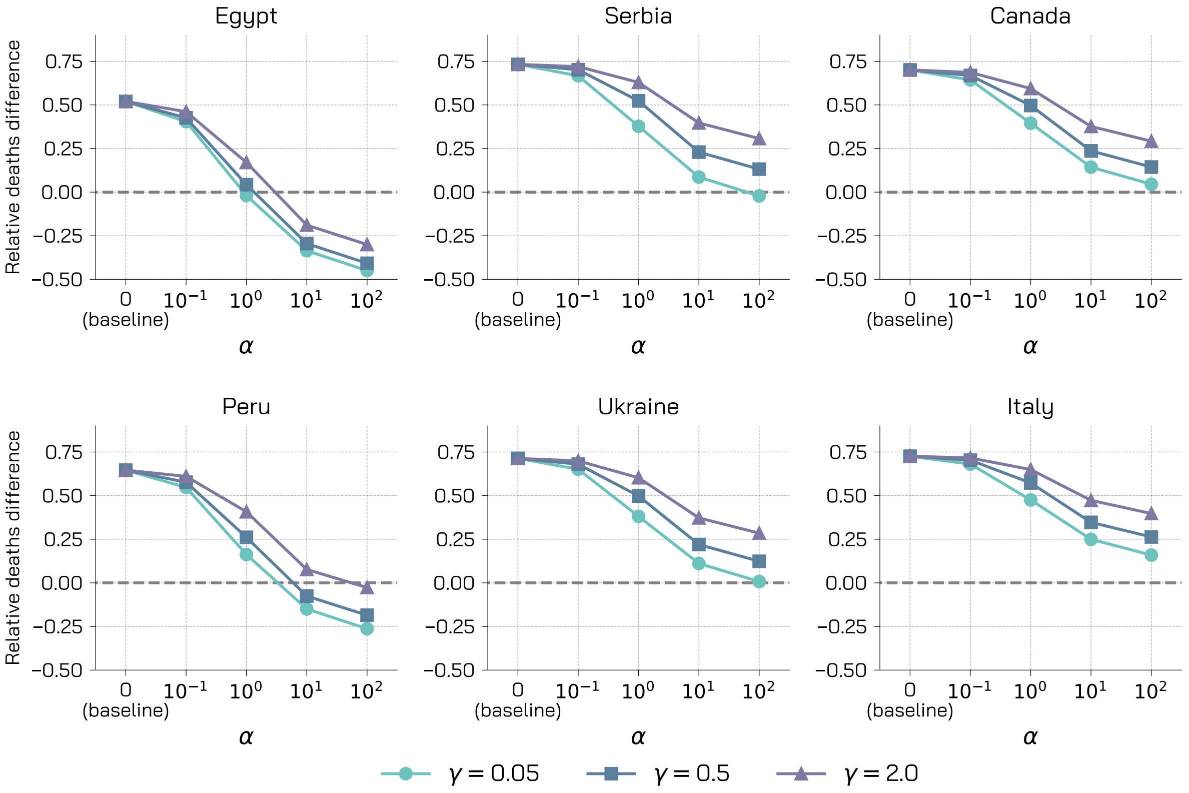

Fig 12. Relative deaths difference for different γ. We represent for the different countries the relative deaths difference for three values of the behavioural parameter γ ($\gamma = 0.05, 0.5, 2$). We consider vaccination strategies prioritizing age groups $20-49$. We set $R_0 = 1.15$, $r = 1.5$, $r_V = 1\%$, $VE_S = 70\%$ ($VE = 90\%$), 0.5% of initially infected, 10% of initially immune individuals, and simulations length is set to 1 year.

2.4 Double Behavioural Rate Model: α_S and α_V

S and V individuals may give up COVID-safe behaviours for different reasons and as result show different behavioural rates. In the main text, however, to avoid complicating further the model, we assumed that S and V individuals can transit to non-compliance (and back) at the same rates, α and γ. Here, we extend the modeling framework to account for heterogeneous behavioural rates. We introduce α_S and γ_S for susceptibles and α_V and γ_V for vaccinated. In Fig 13 we explore the phase space of the behavioural parameters (α_S, α_V) in terms of relative deaths difference with respect to a baseline without vaccine and no behaviour response. For simplicity we keep $\gamma_S, \gamma_V = 0.5$. We consider two vaccine efficacy $VE = 50\%$ ($VE_S = 30\%$) and $VE = 90\%$ ($VE = 70\%$). In general, we observe that the relative deaths difference is more influenced by α_S rather than α_V. Indeed, thanks to the protection guaranteed by the vaccine, the behaviour relaxation of V individuals leads to less infections with respect to a similar reaction from S. This become less evident when lower vaccine efficacy are considered. Indeed, a lower protection expose V individuals to an infection risk which becomes increasingly more comparable to that of S.

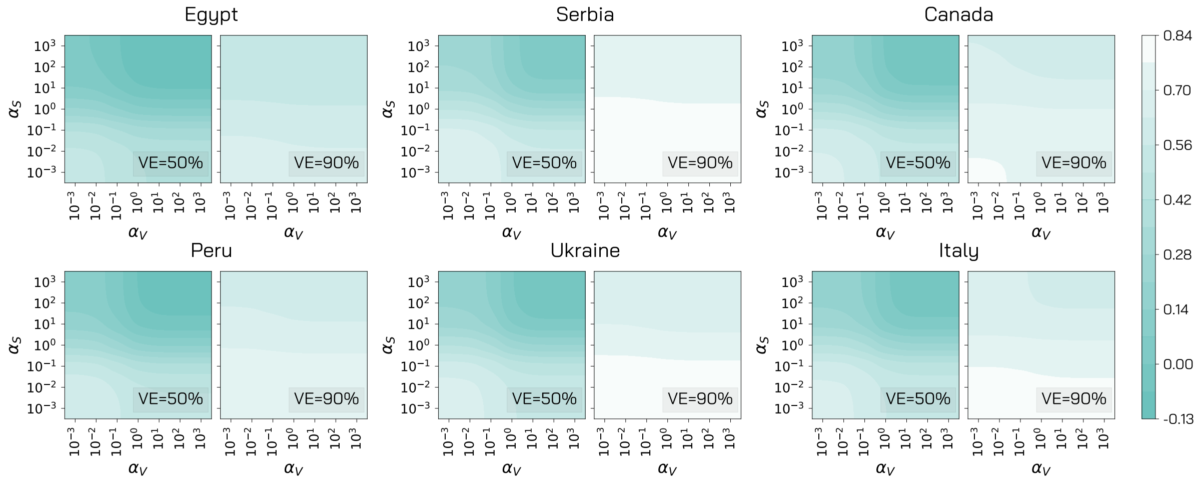

Fig 13. Impact of behavioural parameters α_S and α_V governing the behavioural response. For the six countries, we explore different values of α_S and α_V in terms of fraction of averted deaths with respect to a baseline without vaccine and no behavioural response. For simplicity we keep $\gamma_S, \gamma_V = 0.5$. We consider two different values of the vaccine efficacy $VE = 50\%$ ($VE_S = 30\%$) and $VE = 90\%$ ($VE = 70\%$) and we employ vaccination strategy 1. We set $R_0 = 1.15$, $r = 1.3$, $r_V = 1\%$, 0.5% of initially infected, 10% of initially immune individuals, and simulations length is set to 1 year.

3 Calibrated Model

3.1 Posterior Distributions

In Fig 14 we plot, for the different countries, the posterior distributions of the parameters calibrated through the Approximate Bayesian Computation rejection algorithm. In particular, we display the posterior distribution for the transmission parameter β, the initial number of infected individuals per $100,000$ individuals (split between the L, P, I, and A compartments), and the delay in deaths Δ (i.e., the number of days between the transitions $R_I \to D$ and $D \to D^o$).

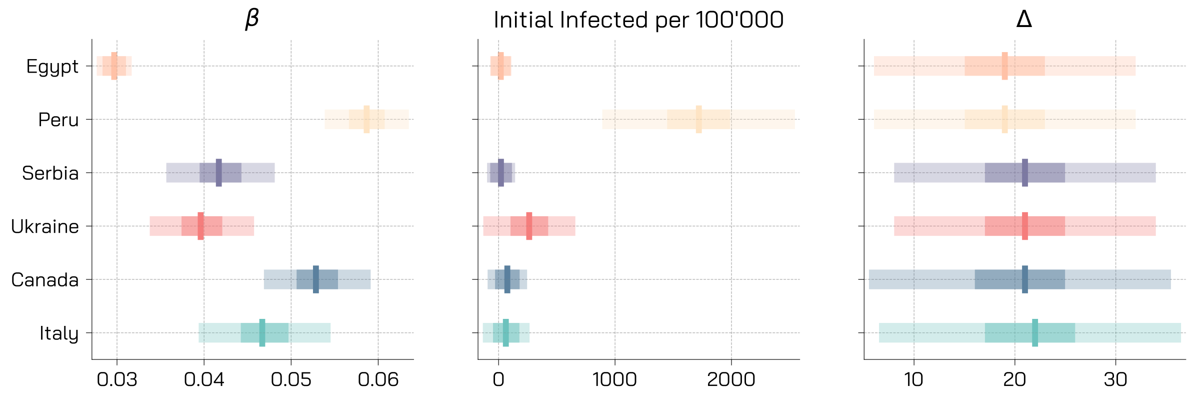

Fig 14. Posterior Distributions. We represent with boxplots the posterior distribution of the calibrated parameters for the different countries. Solid vertical lines indicate the median. The bounds of the darker shaded area indicate the first and third quartile, $Q1$, $Q3$. Their difference is defined as interquartile range $IQR = Q3 - Q1$. The bounds of the lighter shaded area indicate the "minimum" and the "maximum", defined respectively as $Q1 - 1.5IQR$ and $Q3 + 1.5IQR$.

3.2 Robustness of Vaccination Strategies to Behavioural Change

In the main text we introduced the quantity $\Delta_{RDD}(\alpha) = RDD(\alpha) - RDD(0)$. This represents the fraction of averted deaths that are lost because of behaviour relaxation. An analogous quantity can be computed for infections (Δ_{RID}). We use this metrics in Fig 15 to compare, for the calibrated model, the different strategies in terms of robustness to behaviour change. We observe that strategy 1, aimed at reducing severity, is always the more robust to NPIs relaxation when considering averted deaths. When considering averted infections, instead, strategy 2 (homogeneous) and 3 (aimed at reducing transmission) are generally preferable, with the exception of Canada, where also in this case strategy 1 is slightly more robust.

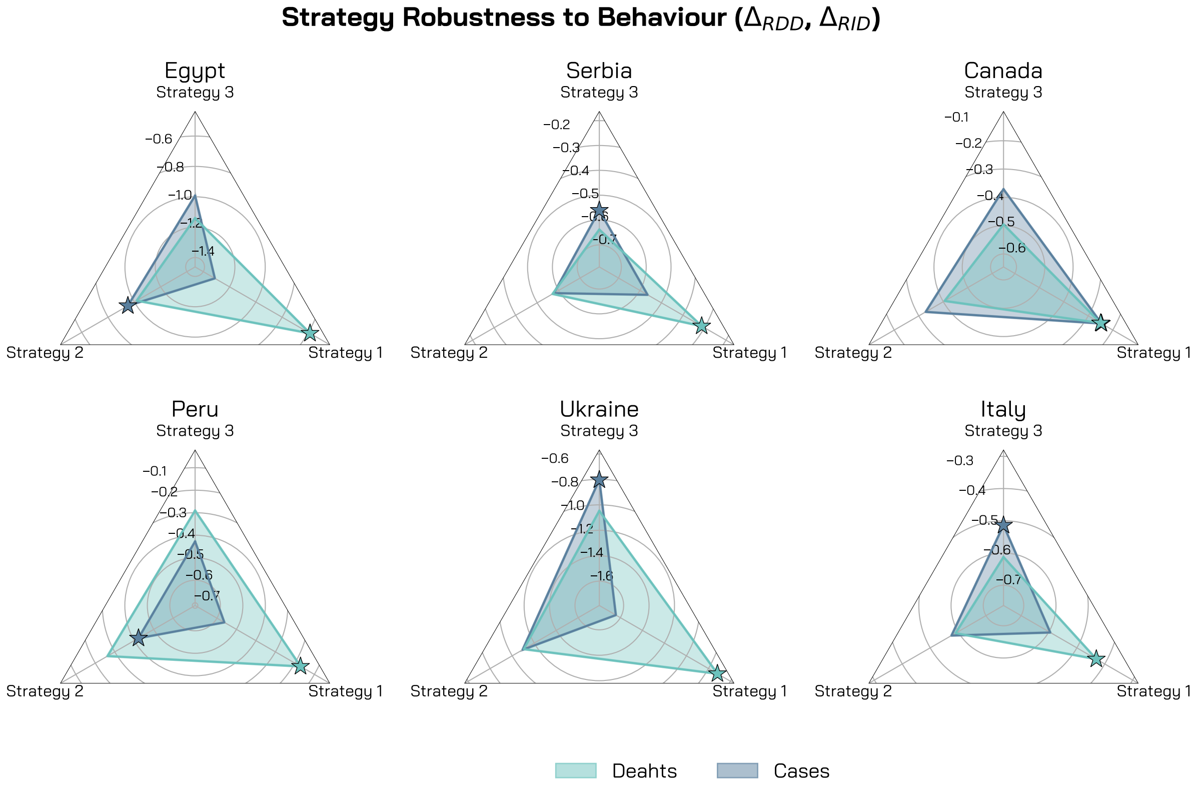

Fig 15. Comparison of different vaccination strategies in terms of robustness to behaviour change. We compare the three vaccination strategies considered in terms of robustness to COVID-safe behaviours relaxation considering both averted deaths and averted infections. Stars indicate best strategy. We set $\gamma = 0.5$, $R_0 = 1.15$, $r = 1.3$, $r_V = 0.5\%$, $VE_S = 70\%$ ($VE = 90\%$), 0.5% of initially infected, 10% of initially immune individuals, and simulations length is set to 1 year.

3.3 Real Vaccination Rollout

Here, we extend the results presented in the main text for the calibrated model integrating also real data from an ongoing vaccination campaign. Because of data availability at the moment of writing, for this analysis, we will focus only on the case of Italy. For the sake of simplicity and to match the available data with the modeling framework presented in this work, we consider only first doses administered daily to individuals in the different age brackets from Ref. [7]. In doing so, we have a data-driven, time-varying estimate of the vaccine rollout speed r_V and of the prioritization strategy. At the bottom of Fig 16 we show the evolution in the first six months of 2021 of the cumulative percentage of people who received at least one dose. During this period, Italy managed to deliver at least one dose to roughly 40% of the population. In the figure, we also show the distribution of doses among the different age groups. Around 91% of the population aged over 75 received at least one dose, but we observe high percentages also in younger groups (for example around 70% in the population aged $60-75$.)

We consider the same calibration step presented in the main text which takes into account real epidemiological and mobility from Ref. [8, 9] during the period 2020/09/01 − 2020/12/31 to set the values of the free parameters of the model. After the calibration, we simulate the unfolding of the epidemic, of the restrictions, and of the vaccination campaign using the real rollout data between 2021/01/01 and 2021/06/01. At the top of Fig 16, we plot the relative deaths difference for a spectrum of α values and for two values of the parameter r capturing the increased risk of non-compliant individuals. Also, when considering real data on the rollout progression, our findings are qualitatively similar to those obtained in the main text. Indeed, we observe that, while with an $\alpha = 0$ the fraction of averted deaths is about 18%, this fraction lowers and turns negative for higher values of α. As expected, the higher value of r leads to worse outcomes.

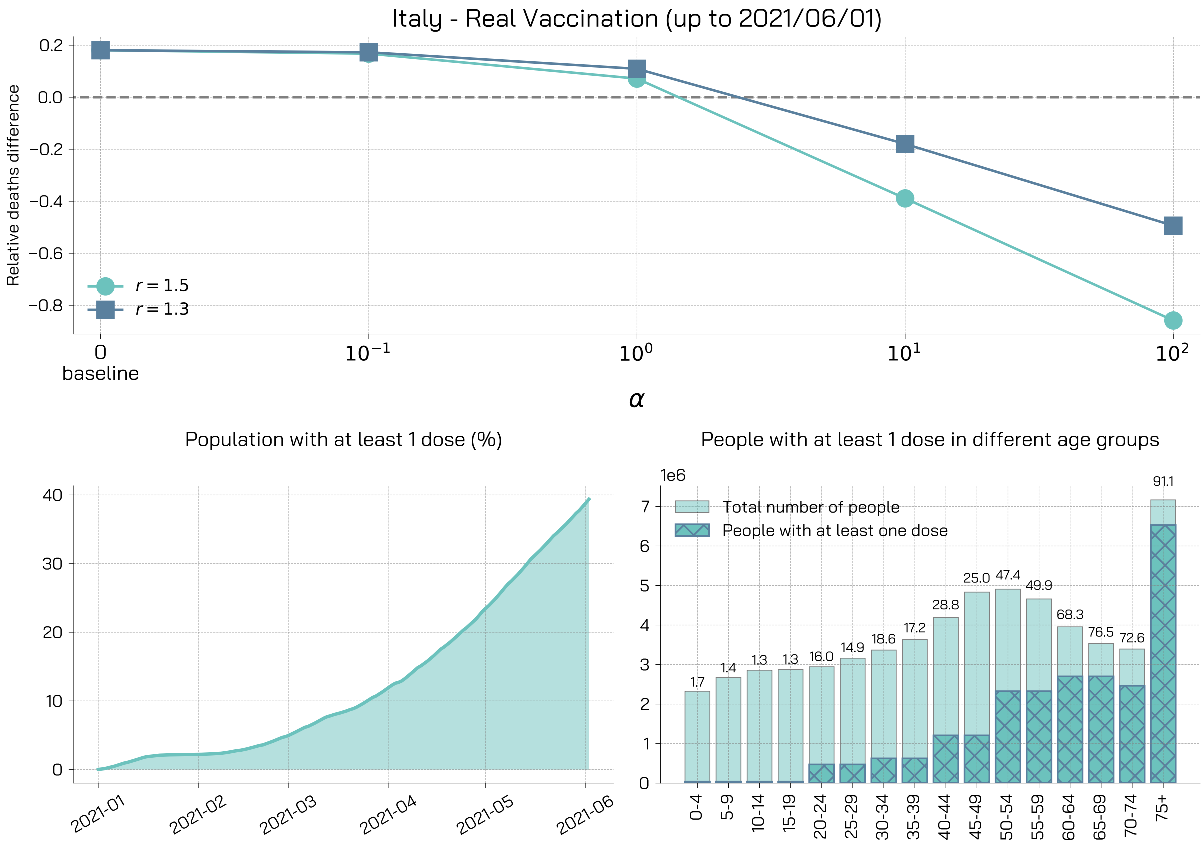

Fig 16. Calibrated model with real vaccinations data.

3.4 Reopening Scenarios

In the main text, we used the Google Mobility Report [8] and the Oxford Coronavirus Government Response Tracker [9] to inform contacts reduction over time up to week 11, 2021. Afterwards, we kept contacts at the level observed for week 11.

Here, we propose a different reopening scenario in which we use the real mobility and policy data for the whole simulation period (2021/01/01 - 2021/06/01).

We repeat the analysis presented in the main text with this new setting for contacts reduction and we display results in Fig 17. In panel A, we observe that after week 11 (indicated with a vertical black line) contacts follow different patterns across the countries under study. Overall, we observe a partial ease of the measures since May 2021. In panel B, we report the quantity Δ_{RDD} (i.e., additional fraction of deaths occurred because of behaviour change) for the three vaccination strategies and the two rollout speeds using the new contacts reduction parameters in the simulations. We observe similar trends to those obtained in the main text. The faster rollout is indeed much more robust to COVID-safe behaviours relaxation. With the slower rollout, on the other hand, much more deaths that would have been averted thanks to the vaccine, occur because of low compliance to NPIs. Similarly to the results of the main text, we see that Egypt and Ukraine are particularly affected, even though the comparison between countries is more difficult since we are letting vary the contacts reduction parameters over longer period of 6 months.

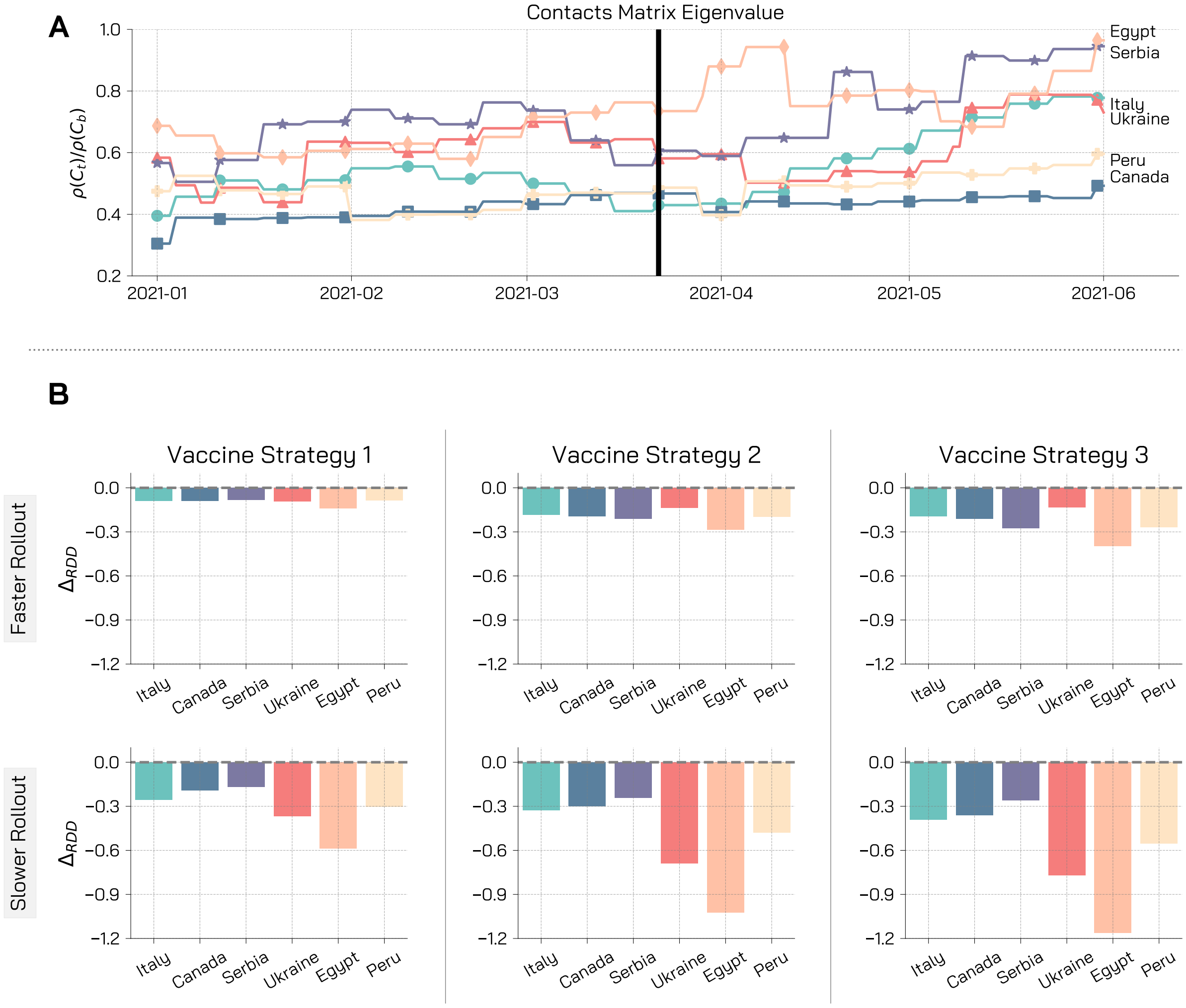

Fig 17. Giving up NPIs during rollout may nullify the benefits brought by the vaccine - Mobility and Policy data up to 2021/06/01. A) We display for the different countries the ratio between the leading eigenvalue of the contacts matrix considering restrictions and of the baseline contacts matrix with no restrictions. B) We display the median relative deaths difference for the calibrated model in the different countries. We consider the three vaccination strategies and two possible rollout speed: $r_V = 1\%$ (faster rollout), and $r_V = 0.25\%$ (slower rollout). We run the model over the period 2021/01/01-2021/06/01. Other parameters are $\gamma = 0.5$, $r = 1.3$, $VE_S = 70\%$ ($VE = 90\%$).

References

1. Driessche P. Reproduction numbers of infectious disease models. Infectious Disease Modelling. 2017;2. doi:10.1016/j.idm.2017.06.002.

2. Lazarus JV, Ratzan SC, Palayew A, Gostin LO, Larson HJ, Rabin K, et al. A global survey of potential acceptance of a COVID-19 vaccine. Nature medicine. 2020; p. 1–4.

3. Liang M, Gao L, Cheng C, Zhou Q, Uy JP, Heiner K, et al. Efficacy of face mask in preventing respiratory virus transmission: A systematic review and meta-analysis. Travel Medicine and Infectious Disease. 2020;36:101751. doi:https://doi.org/10.1016/j.tmaid.2020.101751.

4. Yang B, Huang AT, Garcia-Carreras B, Hart WE, Staid A, Hitchings MDT, et al. Effect of specific non-pharmaceutical intervention policies on SARS-CoV-2 transmission in the counties of the United States. Nature Communications. 2021;12(1):3560. doi:10.1038/s41467-021-23865-8.

5. Mitze T, Kosfeld R, Rode J, Wälde K. Face masks considerably reduce COVID-19 cases in Germany. Proceedings of the National Academy of Sciences. 2020;117(51):32293–32301. doi:10.1073/pnas.2015954117.

6. Haug N, Geyrhofer L, Londei A, Dervic E, Desvars-Larrive A, Loreto V, et al. Ranking the effectiveness of worldwide COVID-19 government interventions. Nature Human Behavior. 2020;.

7. Covid-19 Opendata Vaccini, Italy; 2021. `https://github.com/italia/covid19-opendata-vaccini`.

8. Google LLC "Google COVID-19 Community Mobility Reports"; 2020. `https://www.google.com/covid19/mobility/`.

9. Oxford COVID-19 Government Response Tracker; 2020. `https://www.bsg.ox.ac.uk/research/research-projects/coronavirus-government-response-tracker#data`.
