# Supplementary material for: The importance of non-pharmaceutical interventions during the COVID-19 vaccine rollout
Source: PLoS Comput Biol. 2021 Sep 10;17(9):e1009346. doi: 10.1371/journal.pcbi.1009346 (PMC8457458; doi:10.1371/journal.pcbi.1009346)
Supplement: Attachment — Submitted filename: Gozzi_response_to_reviewers.pdf [file pcbi.1009346.s002.pdf]

Dear Editor,

Please find enclosed a revised version of the manuscript entitled ``The importance of non-pharmaceutical interventions during the COVID-19 vaccine rollout'', which we would like to resubmit for publication in PLOS Computational Biology.

We are extremely happy to see that the reviewers consider the work presented as “interesting” and “an important contribution to the field”. We acknowledge however the doubts and concerns raised by both.

We took all suggestions, comments, and criticisms into serious consideration and revised the paper accordingly.

While we report a point-by-point response to the reviewers below, this is a summary of the changes we implemented:

- we have streamlined the narrative condensing the first part of the paper
- we have changed the implementation of vaccinations making it more realistic
- we provide additional justification about the range of some key parameters
- we have conducted several sensitivity analyses further exploring assumptions and parameters
- we now provide a detailed description of the effect of the behavioral response on several epidemiological compartments

Following reviewers suggestions, we have also realized several additional analyses now featured in the Supplementary Information:

- We extended the modeling framework to include vaccine hesitancy (i.e., fraction of the population refusing the vaccine)
- We provided additional information about the demographics of different countries (layers of contacts matrices, population pyramids) and a practical example comparing Italy and Egypt to further highlight the interplay among socio-demographic features, vaccines rollout, and behaviour change
- We explored a different formulation of the model in which vaccinated and susceptible individuals can change behaviour at different rates
- We extended the exploration of the phase space of the model testing a wider range of parameters.

We dare to hope that this second version of the manuscript adheres to the stringent standards of PLOS Computational Biology.

We thank you in advance for your consideration.

Sincerely yours,

N. Gozzi, P. Bajardi, N. Perra

Reviewer #1:

We would like to thank the reviewer for the interesting comments and constructive criticisms that really helped us improve the manuscript.

Below we report point-by-point answers.

The paper by Gozzi et al attempts to understand how vaccination rates interact with changing population behavior to impact the course of the COVID-19 pandemic. They develop a novel mathematical model that incorporates behavioral changes into traditional age-structured dynamics, and compare pandemic trajectories across a wide-range of parameter values. Overall, their model presents an interesting finding that vaccination can actually cause pandemic surges if populations relax too quickly alongside the rollout. While I believe this to be an interesting and important contribution to the field, I have a number of concerns that I believe should be addressed before publication.

We are extremely happy to see that the reviewer considers our work interesting and an important contribution to the field. We took into consideration all the comments and suggestions.

Major Comments

1) Overall, I believe there is a bit of redundancy in the manuscript, and I would suggest that the authors work hard to reduce the word count and/or reduce the figure count. For example, figures 1 through 4 alongside their respective descriptions in the results present many of the same conclusions, but with slight differences. To improve clarity, I would suggest attempting to focus some of the results around the main conclusions with some of the intricacies added to the supplemental information. Perhaps, even, the manuscript could focus solely on exploring the calibrated model results, and the simulated results could be added to the supplement and summarized briefly.

We discussed at length about removing the first part of the paper and focus entirely on the calibrated model. However, we feel that the first part is key to dissecting the complexities and interactions between the different parameters and characteristics of the countries (i.e., contact matrices, age pyramids). In fact, the calibration fits each country to the specific epidemic conditions which are drastically different across the board.

However, we agree with the reviewer that reducing the number of figures and the description of the first part would help the narrative as well as highlight the results. To this end, we have moved some of the figures and corresponding discussion in the SM. In particular, Figure 1 now features the relative difference in terms of deaths as a function of alpha for different rollout speeds. We moved the equivalent plot for different vaccine efficacy in the SM. We have also moved Figure 5 (radar plot) and Figure 7 (phase space) of the previous version in the SM.

2) While I understand that it is important to not needlessly develop overly complex mathematical models, I believe the authors have oversimplified the vaccination process, which may impact their overall conclusions. First, the authors only allow for susceptible people in their model to be vaccinated. Realistically, many vaccines go to people who have already been infected and are recovered. This could change the relative dynamics between countries with varying levels of infection-derived immunities.

We agree with the reviewer. The initial model was probably too simple regarding vaccine implementation. In the revised version we compute the effective number of vaccines available to susceptibles taking into account that also latent, pre-symptomatic, infectious asymptomatic, and recovered asymptomatic (L, P, A, R_A) can receive the vaccine. We administer the effective number of doses to the susceptible individuals (S, S_NC) and we assume that the other doses are wasted.

This approach has been used in two influential papers in the context of modeling COVID-19 vaccination campaigns:

Matrajt, L., Eaton, J., Leung, T., Brown, E. R., Vaccine optimization for COVID-19: Who to vaccinate first?
Science Advances03 Feb 2021 : eabf1374

Matrajt, L., Eaton, J., Leung, T. et al. Optimizing vaccine allocation for COVID-19 vaccines shows the potential role of single-dose vaccination. Nat Commun 12, 3449 (2021). https://doi.org/10.1038/s41467-021-23761-1

3) Second, as the authors note, there is strong evidence that the vaccines prevent severe outcomes from breakthrough infections. The current model structure treats vaccine breakthrough infections similarly to traditional infections. Altering this to more accurately capture the vaccine protection could decrease the overall mortality rates in the simulations, but it might have larger impacts at different levels of alpha, so I think it would be worth investigating.

Absolutely a good point. We acknowledge that our approach simplifies the effect of vaccination on epidemic trajectories, yet it is widely used (as in the recent Matrajt et al. "Vaccine optimization for COVID-19: who to vaccinate first?." Sci. Adv. (2021) and the leaky formulation in Bubar et al. "Model-informed COVID-19 vaccine prioritization strategies by age and serostatus." Science (2021)). In the revised version of the manuscript we modified the compartmental structure in order to represent the vaccine effect in a more realistic way. First, we added specific compartments to follow the disease progression of vaccinated individuals that get infected, namely L_V, P_V, A_V, I_V, RA_V, RI_V. Second, we split the vaccine effect into two contributions VE_S and VE_Symp, where:

- **VE_S: this term represents the reduction in susceptibility of vaccinated individuals. If the force of infection at time t is denoted as λ(t), vaccinated individuals get infected at a lower rate (1 - VE_S) λ(t);**
- **VE_Symp: vaccinated individuals that get infected have lower probability of developing symptoms and of facing severe outcomes such as hospitalization or death. We include this aspect reducing the probability of being symptomatic for P_V individuals as (1 - f) (1 - VE_Symp).**

This new formulation implies that the overall efficacy of vaccines against severe outcomes such as death in our simulation is given by:

VE= 1- (1-VES)(1-VE_Symp)

This approach to model vaccine effects has been used in:

Matrajt, L., Eaton, J., Leung, T. et al. Optimizing vaccine allocation for COVID-19 vaccines shows the potential role of single-dose vaccination. Nat Commun 12, 3449 (2021). https://doi.org/10.1038/s41467-021-23761-1

And as a sensitivity check in the Supplementary Material of:

Matrajt, L., Eaton, J., Leung, T., Brown, E. R., Vaccine optimization for COVID-19: Who to vaccinate first?
Science Advances03 Feb 2021 : eabf1374

We find that, with this new approach, the overall trends remain unchanged.

4) The authors model noncompliance rates of vaccinated and unvaccinated individuals with the same value, and don't seem to explore scenarios where vaccinated individuals are more likely to be noncompliant than unvaccinated individuals. I think it would be interesting, and more realistic to think about scenarios where noncompliance rates differ between vaccinated and unvaccinated individuals, and suggest that the authors carry out a sensitivity analysis to understand the dynamics or provide more justification for that assumption in their model.

This is a very interesting suggestion. We have added in SM a sensitivity analysis on this choice. We introduce two different behavioural parameters, alpha_S for susceptibles and alpha_V for vaccinated. We explore the phase space (alpha_S, alpha_V) for two different vaccine efficacy. We observe that, in general, alpha_S is more important. Indeed, vaccine protection exposes non-compliant vaccinated people to a lower risk of infection with respect to non-compliant susceptible people. As expected, we also observe that when a lower vaccine efficacy is taken into account, alpha_V becomes more important. Indeed, the lower protection guaranteed by the vaccine exposes non-compliant vaccinated people to an infection risk which becomes more similar to that of non-compliant susceptibles.

5) Given the novel model structure and implementation of noncompliance behavior, I think the authors should add a bit more information about the impact that the behavioral dynamics are having on their results. I believe this to be one of the most interesting aspects of the paper and is underexplored in the results. If the authors do choose to reduce the current figure/word count then I think it would make room for a figure and results exploring the impact the behavioral changes are having. A number of interesting questions could be explored (I am not suggesting exploring all of these, but they're meant to be illustrative of some of the questions I had while reading):

How do the behavioral dynamics impact epidemic curves? What fraction of the population is actually becoming noncompliant in the various simulations?

Is there a specific relationship between vaccine efficacy and noncompliance rates that can help us understand the ultimate relative death difference?

We agree with the reviewer on the importance of characterizing more in detail the effects of the behavioral response. We have added an analysis in the SM to investigate more in depth the role of behavioural parameters. The SM now features a figure representing the probability of the behavioural transitions (compliant -> non-compliant and opposite one) for a wide range of alpha and gamma as a function of the fraction of vaccinated and observed daily deaths per 100'000. Following the reviewer suggestion, we have also investigated for different epidemiological conditions (R_0) and values of alpha the temporal evolution of the fraction of NC individuals represented together with the time-varying rates regulating the behavioural transitions.

How do noncompliance rates within the model compare with the real-world?

As mentioned in the introduction there are several surveys and analyses with data proxies such as mobile phones about the compliance to various NPIs. When it comes to noncompliance induced by overconfidence on vaccines the evidence, to the best of our knowledge, is instead still very limited. We have referenced a few studies that provide some estimates in the introduction, but the sample size and settings where these surveys have been conducted make it difficult to generalize. As we write, we are actually designing a data collection via Facebook to answer this critical question, but at this stage much more work is needed to provide a firm answer.

Nevertheless, we would like to stress how our research is a theoretical analysis of NPIs' relaxation induced by vaccination rollout. As such, while we see the value of constraining the model to empirical observations, we think that exploring a wide range of parameters is indeed important.

Following similar concerns of reviewer #2 we added an analysis in the SM on the effects of behavioural parameters on the probability of behavioural transitions and on the epidemic curves.

Minor Comments

While I don’t think it will dramatically impact the results, I think it would be useful to have more justification for the assumption that everyone in an age group gets vaccinated. In most regions we are seeing saturation of vaccination rollout under 100%, so it will be useful to ensure the results hold for those scenarios.

We thank the reviewer for this comment. Indeed, unfortunately vaccination uptake very likely will not reach 100% coverage in any age-bracket. We have added a sensitivity analysis of this point in the SM. In particular, we have explored different rates of vaccine hesitancy. As expected, we find that a lower fraction of the population willing to receive a vaccine leads to worse outcomes in terms of averted deaths. More interestingly, similarly to what we observed for lower rollout speeds and vaccine efficacy, we find that COVID-safe behaviour relaxation affects more significantly higher percentages of vaccine hesitancy.

One aspect that could be interesting to consider is the difference in countries in anticipated vaccination rollout plans. For example do most of the investigated countries have similar numbers of vaccines proportional to their population, or are there large differences between them?

Unfortunately, as mentioned in the main text we expect large differences in rollout speed. While Italy, Canada, Serbia are somewhat comparable, Peru, Egypt and Ukraine are lagging behind. This is the main reason why we decided to investigate different values of the rollout speed r_V. Our aim is indeed to cover the spectrum of vaccine rollout speeds worldwide.

Nonetheless, we considered only the overall vaccination rate, as many countries do not provide reliable disaggregated data describing vaccination rates for different age-brackets. In the SM, we have presented as additional analysis the case of Italy with real vaccination rates and allocation strategies among age brackets.

The authors model the proportion of asymptomatic similarly across all age groups, but there are key differences between age groups in terms of asymptomatic rates. I would suggest changing this assumption to match the data, or justifying it more for the purposes of the study. See: https://www.nature.com/articles/s41591-020-0962-9

While this is an interesting direction, we feel it would complicate even more the modelling approach as well as the phase space of parameters and interpretation of the results. Considering that we are not aiming to make actual predictions/forecasts we leave this for future extensions.

Since most countries are not homogeneously distributing vaccines, I thought it might be more realistic to have a different realistic scenario that is a middle ground between prioritizing the eldest age groups and the highest transmitting age groups: splitting vaccine prioritization between the high spreaders and high mortality age groups at the same time.

The reviewer is absolutely right. Real vaccination rollouts do not strictly follow any of these “pure” strategies. In fact, we have explored the case of Italy using the real data in the SM. As shown, while there is a clear prioritization of the ederly, in the initial phases of the campaign medical personnel (irrespective of age) have been vaccinated. We feel that adding a fourth mixed strategy could increase the complexity of the figures and the discussion. Hence, we have opted to leave this to future work and to highlight more clearly the case of Italy presented in the SM in the main.

The expressions for g(alpha) and h(gamma) are not clear in the model. For example line 566 is different from line 514, and their relationships are unclear.

The reviewer is right. However, the mismatch between the expression reported in the previous version at line 514 and 566 arises from the fact that g(alpha) and h(gamma) are just general terms whose expression depends on the specific mechanism employed to model behavioural change. Nonetheless we agree with the reviewer that the previous version was not clear enough on this point. In the “Model Definition” subsection of the revised version we added the explicit expressions for g(alpha) and h(gamma) in the two behavioural mechanisms.

While I believe all of the assumed parameter values are provided for the model described in equation 1, I think it would be useful to have a supplementary table that outlines all of the parameters used in the model that are and are not fitted to data (the model calibration explanation of parameters is extremely clear for those that are fitted).

Great suggestion echoed also by reviewer #2. We have added the table as suggested.

While the paper links to the contact matrices used for their age groups, I think it would be useful to outline what age groups are investigated in the model particularly because the vaccine prioritization strategies are age-based

We use 16 five-year age groups (from 0-5 onwards), with the last one including 75+ individuals. We acknowledge that this information was only mentioned briefly in the subsection “Model Initialization”. To improve clarity we stressed the age group division also in the “Model Definition” subsection, and added a dedicated section in the Supplementary Information to better describe the

population pyramids and contact matrices of the countries under investigation.

Is there any justification for choosing r = 1.3 or r = 1.5? It might be useful to think about other forms of noncompliance (e.g. masking behavior), and the impact they had on transmission dynamics in justifying these values.

Following similar concerns of reviewer #2 we have scanned the recent literature aimed at quantifying the impact of different NPIs on transmission to justify and provide sensible/possible ranges for r. In particular, a meta-analysis conducted by Liang, et al. ("Efficacy of face mask in preventing respiratory virus transmission: A systematic review and meta-analysis") suggested that mask use have a significant protective effect on the spread of respiratory viruses (OR = 0.35 and 95% CI = 0.24–0.51).

More recently, a study conducted in the US by Bingyi et al. ("Effect of specific non-pharmaceutical intervention policies on SARS-CoV-2 transmission in the counties of the United States.") reported that "Stay at home orders were associated with a 15% reduction in the effective basic reproductive number (95% CI 13 to 17%) while face-mask orders were associated with a 18% reduction (95% CI 16% to 20%).". Similarly, Mitze et al. ("Face masks considerably reduce COVID-19 cases in Germany.") found that face masks reduced the number of newly registered severe acute respiratory syndrome coronavirus 2 infections between 15% and 75%.

Nils et al. ("Ranking the effectiveness of worldwide COVID-19 government interventions.") analysed the effect of several NPI, finding that the largest impacts on Rt are shown by small gathering cancellations (83%, ΔRt between −0.22 and –0.35).

While it is very hard to estimate the effect of specific NPIs and general covid-safe behaviours, it is also quite difficult to translate different estimates into model parameters. Hence, we choose to let the parameters change within a reasonable ballpark informed by the aforementioned papers. We have added a brief discussion in the main text regarding the values chosen for the parameter r.

The authors don't discuss the potential impact of testing on the ultimate epidemic trajectories. It might be interesting to consider the relative testing capacities of the countries, and what impacts they could have on the pandemic.

As mentioned above in the point about differences in asymptomatic prevalence across age-brackets, we feel that including testing would increase the complexity of the model. Furthermore, it is important to stress that

reproducing in detail the pandemic trajectory of each country is not the aim of our research.

In lines 250 to 252, it is stated that: “Moreover, it is worth stressing that these results are to be intended in relative terms: a relative worst performance in averting deaths does not necessarily imply a worst absolute performance”. Could the authors please explain what they mean related to absolute performance. If there are important differences, I would suggest including a supplemental figure that demonstrates this dynamic.

What we meant is that a relative worst performance might not translate into a drastic absolute difference. In certain regions of the phase space the number of deaths is very limited, so an increase of 20% of deaths might translate into a few more deaths in total. We have revised the text to make it more clear. We have also added a clarifying example in the SM (in the “Demographic” section) in which we compare Italy and Egypt.

Reviewer #2:

We would like to thank the reviewer for all the comments, suggestions and constructive criticisms. They helped us improve the paper.

Below we report point-by-point answers. We highlighted in blue the reviewers’ comments.

In the manuscript entitled “The importance of non-pharmaceutical interventions during the COVID-19 vaccine rollout” Gozzi et al use an aged-structure mathematical model to explore how different key factors (vaccine efficacy, vaccination rate, vaccine allocation, behavioural changes) affect the impact of vaccine rollout in six different countries.

This is an interesting paper with interesting results. While there are a lot of others who have already reached similar conclusions (that NPIs need to be maintained through vaccine rollout) this paper is a nice addition to the existing literature because it attempts to explicitly model the influence of vaccination in human behavior and because it explores all these scenarios in 6 countries where little to no modeling has been done before.

We are extremely happy to see that the referee finds our paper and results interesting. We are also happy to see that the referee appreciated our conscious decision to explore a wide range of countries besides those widely studied in the literature.

Major concerns

1. While this modeling of human behavior is indeed attractive, it also is where my major concern is. The authors model this through a parameter alpha that ranges from 0 to 1000. This parameter modulates the fraction of susceptibles or vaccinated individuals moving to the classes with increased risk-behavior. There is no rationale or convincing argument as of why/how this parameter was chosen, or why the functional form using this parameter was chosen. The only reference given for this choice is yet another modeling paper, that was not based on data. In fact, the functional form for g(\alpha) is not explicitly given in the paper. Furthermore, the authors state that people in those compartments will have an increased infection probability (r = 1.3 or 1.5). This corresponds to a 30% or 50% increase in transmission for these groups. Again, what is the rationale for these values? Are these values based in some knowledge?

We agree with the referee that some parameters range needed more justification. Following similar concerns of reviewer #1 we have done the following.

First, in the SM we added an analysis to better grasp the role and the effects of the behavioural parameters alpha and gamma. More in detail, we investigate the probability of behavioural transitions N -> NC and NC -> N for a wide range of the parameters alpha and gamma, the fraction of the population vaccinated (v_t) and the observed daily deaths per 100'000 (d^o_t-1). Together with this information, we study the temporal evolution of NC individuals (S and S^NC) for different epidemiological conditions (R_0) and values of alpha.

Second, we have scanned the recent literature aimed at quantifying the impact of different NPIs on transmission to justify and provide sensible/possible ranges for r.

In particular, a meta-analysis conducted by Liang, et al. ("Efficacy of face mask in preventing respiratory virus transmission: A systematic review and meta-analysis") suggested that mask use have a significant protective effect on the spread of respiratory viruses (OR = 0.35 and 95% CI = 0.24–0.51).

More recently, a study conducted in the US by Bingyi et al. ("Effect of specific non-pharmaceutical intervention policies on SARS-CoV-2 transmission in the counties of the United States.") reported that "Stay at home orders were associated with a 15% reduction in the effective basic reproductive number (95% CI 13 to 17%) while face-mask orders were associated with a 18% reduction (95% CI 16% to 20%).". Similarly, Mitze et al. ("Face masks considerably reduce COVID-19 cases in Germany.") found that face masks reduced the number of newly registered severe acute respiratory syndrome coronavirus 2 infections between 15% and 75%.

Nils et al. ("Ranking the effectiveness of worldwide COVID-19 government interventions.") analysed the effect of several NPI, finding that the largest impacts on Rt are shown by small gathering cancellations (83%, ΔRt between −0.22 and –0.35).

While it is very hard to estimate the effect of specific NPIs and general covid-safe behaviours, it is also quite difficult to translate different estimates into model parameters. Hence, we choose to let the parameters change within a reasonable ballpark informed by the aforementioned papers. We have added a brief discussion in the main text regarding the values chosen for the parameter r. We would like to stress how we consider the exploration of wide ranges of such parameters important to theoretically explore the different regions of the model proposed.

The main conclusions of the paper, that with high values of alpha the impact of behavioral change might even produce more deaths, need to be more quantified and put in context with what these parameters are doing. For instance, for a rate of \alpha > 10 and slow vaccination rate, there is a potential loss of 1.5.

What does alpha > 10 mean? It would be helpful to see what percentage of the compartments (susceptibles or vaccinated) is transitioning to the NC compartments with different values of \alpha to see if these values of \alpha make sense.

As mentioned in the previous point, we have added an analysis in the SM to better understand the effects of behavioural parameters. We study the actual transition probability N -> NC for several values of alpha, the fraction of vaccinated individuals and the fraction of NC individuals in time for different R0 and alpha.

As the paper stands right now, it is not relatable to a wider audience or to public health officials. It would have much more impact if the authors can place their parameters in a context that is understandable by decision makers.

It is important to stress how, admittedly, our results are still too theoretical to directly inform public health officials. The aim is to investigate the possible effects of NPIs relaxation induced by overconfidence on the vaccine showing the peril/ fragility of the initial phases of the rollout. Our results highlight the importance of NPIs adoption as we transition to a new phase of the pandemic. As more data is collected and we move forward with the vaccination rollout, we will be able to follow up this first contribution providing constraints and more direct implications for the pandemic evolution.

Nevertheless, we think that the work we have done in the revision to ground the key behavioral parameters to observations makes our results closer to the ambition of informing the policy cycle. We thank again both referees for suggesting this direction.

Is a loss of 1.5 equivalent to say that there is an increase of 50% more deaths compared to the non-vaccination scenario? This is unclear to me. If this is the correct interpretation of the numbers in Figs 1 and 2 it would be very helpful for the reader to put the results in these terms rather than potential losses (potential loss of 0.21 l228).

We thank the reviewer for highlighting the confusion around this point. In the example mentioned by the reviewer, a loss of 1.5 would imply a 150% increase in the number of observed deaths. Indeed, the relative deaths difference is defined as the fraction of averted deaths in the case of vaccine (and behaviour response) with respect to the case without vaccine (and no behaviour response). To improve clarity, we added the subsection "Relative deaths difference" in the "Material and Methods" section where we report the definition of the metrics used to evaluate the impact of vaccines and behaviour.

2. Overall, I think the paper would benefit of rewriting the results in a more organized and compact way. Right now, there are results in the results section, in the SM and even in the methods section (eg line 547)

The first part of the paper really deals with the problem of how changes VE, vaccination rates and behavior would affect vaccination campaigns for 6 countries with different population structures, with all the other parameters fixed. The important comparisons then should reside in trying to explain how the population structure is driving the results, and I believe that more can be said in that topic, as right now there is only a vague sentence trying to explain this (l 248). It might be good to plot which age group is moving to the NC compartments, and which age groups are dying in each country to try to figure out why countries with larger proportion of younger people feared worse.

Following both reviewers' suggestions/comments on this point, we have restructured and streamlined the first part of the paper.

We have also modified the methods section, trying to shift all the main results in the results section and in the SM.

We thank the reviewer for the interesting suggestions for further analyses. The fraction of people in different age groups transitioning to the NC compartments is solely determined by alpha/gamma and is constant across age brackets. We reasoned about introducing age-dependent alpha/gamma but we decided to leave this for the future when the data we're starting to collect to validate the model and inform the parameters we'll be available. Similarly, also the age groups that are facing worse outcomes (i.e., death) is determined by the infection fatality rate, which we assume to be constant across countries. As we noted in the text, this is a simplifying assumption since we expect variation across health infrastructure and healthcare access in different countries.

Nonetheless, sharing the feeling of the reviewer, we added a section in the SM ("Demographic") to further characterize the sociodemographic differences

between countries and their interplay with vaccines rollout and behaviour change. More in detail, we plot the four layers (school, work, home, other locations) of the contacts matrices for the six countries used in the simulation that we take from:

Prem K, Cook AR, Jit M (2017) Projecting social contact matrices in 152 countries using contact surveys and demographic data. PLOS Computational Biology 13(9): e1005697. https://doi.org/10.1371/journal.pcbi.1005697

We also report the fraction of people in different age groups as reported in:

United Nations, Department of Economic and Social Affairs, Population Division. World Population Prospects: The 2019 Revision; 2020. https://population.un.org/wpp/Download/Metadata/Documentation/

Finally, we have added an interesting example to characterize the impact of behaviour on different demographics. We consider the two most dissimilar countries in our dataset in terms of contact patterns and population pyramids, Italy and Egypt. We run two simulations, one with behaviour change and one without it. We observe that, reasonably, more deaths occur in Italy in both cases: indeed the older population makes the country more vulnerable to the spreading. More interestingly, we observe that in relative terms (%), behaviour change has a much higher impact in Egypt, causing a 59% increase in deaths with respect to the simulation without NPIs relaxation (in Italy we get a 29% increase).

The second part of the paper repeats this exercise with now fitted models to each country. I believe the results with the calibrated models are more interesting, and it might be worth it to put those first. Again, some interpretation of the values of \alpha is really needed here.

We discussed at length about removing the first part of the paper and focus entirely on the calibrated model. However, we feel that the first part is key to dissecting the complexities and interactions between the different parameters and characteristics of the countries (i.e. contact matrices, age pyramids). In fact, the calibration fits each country to the specific epidemic conditions which are drastically different across the board.

However, we have removed some of the figures and corresponding discussion in the first part of the paper in the SM. In particular, Figure 1 now features the relative difference in terms of deaths as a function of alpha for different rollout speed. We moved the equivalent plot done for different vaccine efficacy in the SM.

3. The infection prevalence (number of people currently infected) set at 1% of the population is very high. To date, Canada's worst day ~9,000 new cases. Even if one assumes that only 20% of

infections are reported and that each day there are on average 5x the number of new cases, this comes back to ~ 0.0059 of the population (0.5%) infected.

Our aim in that part of the paper was not to pick a realistic representation of the pandemic for each country. In fact, we tackle that challenge in the second part via a calibration. There, we selected and initialized the spreading for each country in the same conditions. Furthermore, the reviewer is comparing daily incidence with infection prevalence. Indeed, the number of cases reported on a single day is not the number of active cases, and as such is just a fraction of the individuals that are placed in the infected compartments ini our simulations (L, P, I, A). Nonetheless, we agree that 1% is fairly high. In the revised version we used instead 0.5%.

Minor concerns:

1. Fig. 1 is too busy and hard to read. Part A can go to the supplement or split figure 1 into two figures. The middle panel of A is unclear. Does it mean that People in Peru aged 15-19 have >100 more contacts than people in Italy of the same age group? I wonder if there is a better representation of this. Maybe a barplot with the number of contacts for each age group for all 6 countries (one could split it into 2 rows with 8 age groups per row) Part B is too small to note any differences in strategies.

Having in mind the goal to streamline the first part of the paper, figure 1 now features panel B of the previous figure 2. The old panel B of figure 1 has been moved in the SM.

We have changed the middle figure in panel A to clarify the meaning of the y-axis.

2. l.227 “strategy reducing severity” suggest putting in parenthesis the strategy (strategy 1).

That part is now in the SM. We have changed the text to improve clarity.

3. The paper would benefit with a table with the parameters used in the model and a supplemental table with the values obtained by fitting (given in fig4A)

We have added a table as suggested on the parameters used in the simulation. We report the fitted values in a plot of the SM.

4. Figure 5 is not at all clear to me, not clear how the point it is trying to convey is actually conveyed with the figure.

We apologize for the confusion. We have actually changed a bit the quantity represented in that figure, which is now in the SM. We have re-written the description to make it more clear.

5. While the authors cite some of the first papers done in vaccine allocation for COVID-19, there is a vast literature now of papers that addressed very similar issues to what they are addressing (e.g.

https://academic.oup.com/cid/advance-article/doi/10.1093/cid/ciab079/6124429,
https://www.medrxiv.org/content/10.1101/2020.12.31.20249099v4,
https://www.thelancet.com/journals/laninf/article/PIIS1473-3099(21)00143-2/fulltext

https://www.medrxiv.org/content/10.1101/2020.12.30.20248888v2

We thank the referee for the pointers. We have added these references and reframed our narrative accordingly. We have also added some other references to better align our paper with the newly published literature.
